# DNA metabarcoding reveals the dietary composition in the endangered black-faced spoonbill

**DOI:** 10.1038/s41598-021-97337-w

**Published:** 2021-09-21

**Authors:** Pei-Yu Huang, Emily Shui Kei Poon, Anson Tsz Chun Wong, Ivy Wai Yan So, Yik-Hei Sung, Simon Yung Wa Sin

**Affiliations:** 1 School of Biological Sciences, The University of Hong Kong, Pok Fu Lam Road, Hong Kong SAR, China; 2 Wetland and Fauna Conservation Division, Agriculture, Fisheries and Conservation Department, Hong Kong SAR Government, Hong Kong SAR, China; 3 Science Unit, Lingnan University, Tuen Mun, Hong Kong SAR, China

**Keywords:** Conservation biology, Molecular ecology, Conservation biology, Molecular ecology

## Abstract

Extensive loss of natural wetlands caused by changes in land use largely diminishes the food resources essential for the survival of migratory waterbirds. Globally, the decline in waterbird populations in East Asia is the most serious, with 64% of these populations showing a decreasing trend. In this study, we applied DNA metabarcoding to examine the spatiotemporal variations and diversities in the dietary compositions of migratory waterbirds in a natural/artificial wetland complex in Asia. By investigating 110 fecal samples from the endangered black-faced spoonbill (*Platalea minor*) wintering in the wetland, our results show that *P. minor* had a broad dietary spectrum. The birds fed on at least 26 species in the classes Actinopterygii and Malacostraca, with Mugiliformes, Cichliformes, and Gobiiformes being the main taxa in their diets. Our results also demonstrated clear patterns of the spatiotemporal variations between the roosting groups and intraspecific variations between the individuals, which potentially reflect some of their feeding habits, and the probable usage of different habitat types in the wetland complex. Using high-throughput sequencing, we were able to elucidate the food resources that are critical to *P. minor* non-invasively, this method can also be used to provide invaluable information for the conservation of many other waterbird species.

## Introduction

The primary drive behind bird migration is a change in food availability in their habitats, which affects their survival and reproduction of migratory species^1,2^. Wetlands are highly productive and biodiverse ecosystems that serve as breeding, staging, and wintering grounds for migratory waterbirds along the flyways and supply a great variety of food to waterbirds during their long-distance migration^3^. However, wetlands have been facing severe anthropogenic threats and impacts for decades. Natural wetlands were historically regarded as wastelands if not converted into agricultural, aquaculture, industrial, or residential areas^4^. The food web structure of modified wetlands has been extensively impacted by these economic activities, mainly through landscape change and pollution, which progressively decreases the availability of food along flyways and threatens the sustainability of many migratory waterbird populations. Recent figures indicated that more than 50% of wetlands are lost^5^, 55% of the world’s waterbird species are decreasing, and 17% of waterbird species are categorized as globally threatened^6^.

To halt the widespread loss of wetlands, multiple countries have created new or restored/enhanced existing artificial wetlands^7^ as mitigation measures. However, these semi-natural or artificial wetlands in the form of aquaculture ponds or reservoirs, are often functionally different from natural ones and rely on active human management^8^. Newly created or poorly managed wetlands might have less stable communities and lower species abundance and diversity than natural ones, failing to provide suitable food to visiting waterbirds^9,10^. Under the continuous urban population growth, artificial wetlands are expected to expand worldwide as important habitats for waterbirds in the future^5^. Therefore, to optimize wetland management, it is imperative to obtain knowledge about the food resources required for the survival and reproduction of threatened waterbirds.

**Figure 1:**
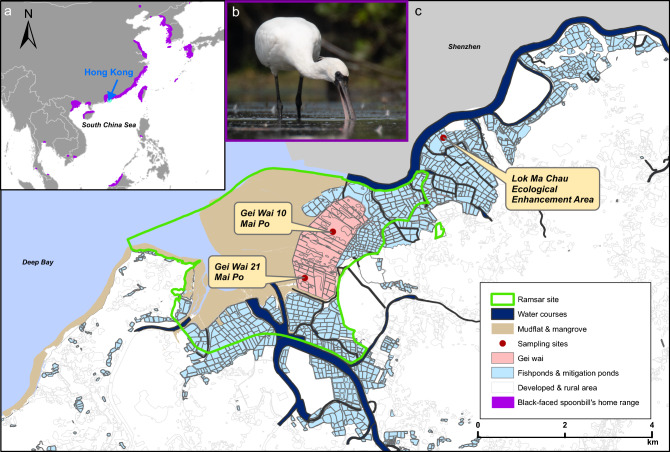
Sampling sites of the wintering black-faced spoonbill (*Platalea minor*). **(a)** Map of East Asia with purple regions indicates the distribution range. **(b)** *Platalea minor* wades to feed near mangroves in the Mai Po Inner Deep Bay Ramsar Site. Photo credit: Hon Shing Fung. **(c)** Map of the northwestern part of Hong Kong where the wetland complex is located. The map was made using ArcGIS version 10.7^70^.

In East Asia, the declines in waterbirds are the most serious among all global regions, with 64% of waterbird species occurring in East Asia showing a decreasing trend. Many of these waterbirds migrate along the East-Asian Australasian Flyway (EAAF)^6^. Hong Kong, one of the many highly developed coastal cities along the EAAF, is located in a central part of the flyway and provides essential wetland habitats for migratory waterbirds^11^. The northwestern part of Hong Kong is a wetland complex where natural, semi-natural, and artificial habitats occur. An important part of this area is the Mai Po Inner Deep Bay Ramsar Site, covering approximately 1500 hectares (ha)^12^. It serves as an overwintering ground for 160 species of over 50,000 migratory waterbirds every year^13^. These include over 300 endangered black-faced spoonbills (*Platalea minor*, Pelecaniformes; Fig. 1), which constitute about 8% of the global population^14^. This Ramsar Site comprises a natural estuarine bay with intertidal mudflats and mangroves, semi-natural tidal shrimp ponds (gei wai), and artificial fishponds^15^. Gei wai is a traditional polyculture practice in which shrimp farmers control the sluice gates to allow naturally occurring shrimp postlarvae and fish fry to migrate from the Inner Deep Bay into the gei wai during the high tide. Artificial fishponds are fish farming systems where fishermen traditionally drain down the ponds for harvesting and refilling to re-stock farmed fish fry^16^. Contiguous to the Ramsar Site, some other large areas of artificial fishponds in this wetland complex cover over 1,000 ha^17^. This internationally important wetland provides us with a unique opportunity to understand the foraging ecology of migratory waterbirds, especially those globally threatened species such as *P. minor*.

The endangered *P. minor* is one of the most iconic species using the EAAF. With a small global population of just 4864 individuals according to the global census in January 2020^18^, *P. minor* is the rarest among all six species of spoonbills worldwide^19,20^. It has been challenging to elucidate the composition of waterbirds' diets accurately, and this especially applies to *P. minor*, which has a specialized feeding strategy. *Platalea minor* is a tactile feeder that forages by wading in the turbid waters of coastal areas, usually at dusk and dawn, sweeping its bill from side to side (Fig. 1b) and swallowing live prey swiftly in a catch-and-throw manner^21^. Traditional methodologies in dietary studies on waterbirds, such as direct observations of feeding behavior and microscopic examinations of the stomach or fecal contents, have many limitations, such as the massive and watery foraging areas, turbidity of waters, swift swallowing movements during feeding, small sizes of food, degradation of digested stomach and fecal contents, which often result in small sampling size and classification of food items into broad taxonomic groups in studies^22^. These limitations reduce the accuracy and effectiveness of estimating the relative proportions of each food component in the diets, as well as the intraspecific and spatiotemporal variations between individual diets, which are very important in revealing the dietary diversity, foraging behavior, and habitat usage of the species. The advent of high-throughput DNA sequencing techniques in recent years offers alternative approaches and advances in the study of animal diets^23^. High-throughput DNA metabarcoding techniques enable the use of genetic markers to characterize the species composition in a complex mixture of fragmented DNA in various environmental samples, such as animal feces, with high taxonomic detectability and resolution^24^. This technique also profoundly facilitates the simultaneous sequencing of samples on a large scale^25^.

Although DNA metabarcoding has already been successfully applied to many dietary studies of wild animals over the last decade^26^, the technique has rarely been applied to globally threatened species^27^, due to the challenge of obtaining fecal samples from rare species with extremely small population sizes. Here, we applied the dietary DNA metabarcoding for the first time to determine the prey composition of endangered *P. minor*. By examining more than 100 individual fecal samples, we elucidated the intraspecific and spatiotemporal variations in the winter dietary spectra of *P. minor*. Specifically, we (1) investigated the prey species of *P. minor* to indicate the food sources essential to the sustainability of its population during the wintering period and (2) examined the differences in dietary compositions between individuals, as well as roosting groups, to unveil the dietary variations and diversities of *P. minor*. This study demonstrates the application of dietary DNA metabarcoding as an important conservation management tool to gain insights into the foraging ecology of endangered species, which can ultimately facilitate the development of effective conservation strategies.

## Results

### Total identified taxa in feces

**Figure 2:**
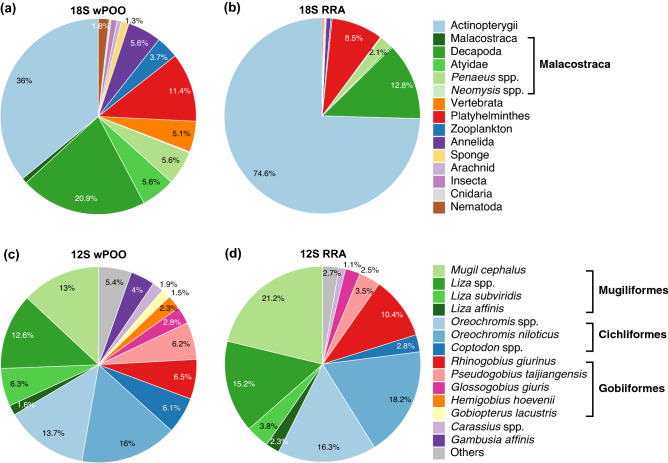
Taxa compositions identified in the fecal samples of *Platalea minor*. Weighted percentage of occurrence (wPOO) and relative read abundance (RRA) of all detected taxa were analyzed using **(a,b)** 18S (n = 110) and **(c,d)** 12S (n = 96) metabarcoding data. Color blocks represent 15 metazoan taxa of 18S rDNA (at or above genus level) summarized from 40 taxonomic groups and 14 major fish taxa (at or above species level). The remaining 15 minor fish taxa (RRA or POO < 1%) were grouped into 'Others'.

The 18S dataset indicated that the class Actinopterygii [74.6% relative read abundance (RRA) and 100% frequency of occurrence (FOO)] and class Malacostraca (15.2% RRA and 73.6% FOO; mainly in the order Decapoda and *Penaeus* spp.) were the most abundant and frequently detected taxa, followed by Platyhelminthes (8.5% RRA and 40.9% FOO; Fig. 2; Supplementary Tables S1 and S2)*.*

Orders Mugiliformes (42.6% RRA), Cichliformes (37.3% RRA), and Gobiiformes (18.1% RRA) accounted for 98% of all 12S reads (Fig. 2d; Supplementary Table S3). Fish species including flathead gray mullet (*Mugil cephalus*), *Liza* spp. (including greenback gray mullet *L. subviridis* and eastern keelback mullet *L. affinis*), and *Oreochromis* spp. (including Nile tilapia *O. niloticus*) were the most abundant (21.2–34.5% RRA and 59.4–66% FOO), followed by gobies (*Rhinogobius giurinus*, *Pseudogobius taijiangensis*, and *Glossogobius giuris*, with 2.5–10.4% RRA and 10.4–32.3% FOO), *Coptodon* spp. (2.8% RRA and 27.1% FOO), and crucian carp *Carassius* spp. (1.1% RRA and 6.3% FOO; Fig. 2d; Supplementary Table S3). We also detected a high diversity of other fish species with low abundances, such as mullets *Valamugil* spp., gobies *Hemigobius hoevenii*, *Gobiopterus lacustris, Mugilogobius abei*, *Mugilogobius chulae*, *Favonigobius gymnauchen*, Eurasian minnow *Phoxinus phoxinus*, topmouth gudgeon *Pseudorasbora parva*, chub *Squalius cephalus*, mosquitofish *Gambusia affinis*, gizzard shad *Nematalosa nasus*, silver sillago *Sillago sihama*, bald glassy *Ambassis gymnocephalus*, bartail flathead *Platycephalus indicus*, and yellowfin seabream *Acanthopagrus latus* (0.008–0.69% RRA; Fig. 2d; Supplementary Table S3).

### Fecal taxa composition of roosting groups and individuals

**Figure 3:**
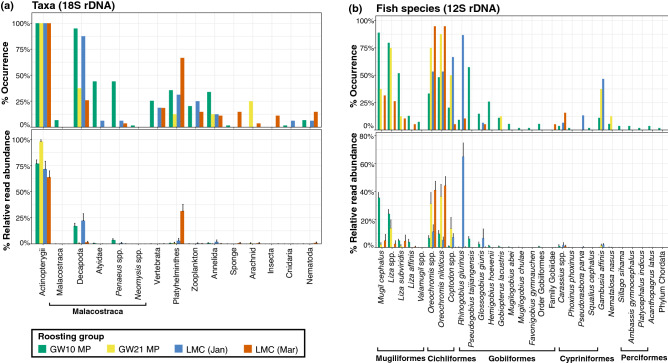
Occurrences and abundances of taxa detected in the fecal samples of *Platalea minor* based on **(a)** 18S rDNA (n = 110) and **(b)** 12S rDNA (n = 96). Frequency of occurrence (upper panel) and means and standard errors of the relative read abundance (lower panel) of each taxon detected in samples from different roosting groups were shown. *GW* gei wai, *MP* Mai Po Marshes and Inner Deep Bay, *LMC* ecological enhancement area of Lok Ma Chau, *Jan* January, *Mar* March.

**Figure 4:**
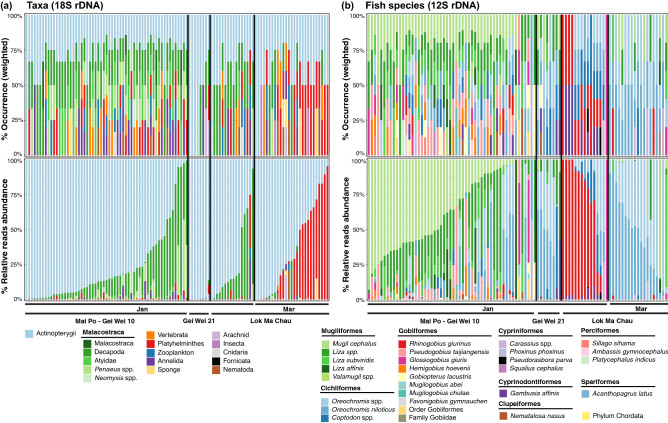
Taxa proportions in the feces of *Platalea minor* detected using the **(a)** 18S rDNA (n = 110) and **(b)** 12S rDNA marker (n = 96). Weighted percentage of occurrence (upper panel) and relative read abundance (lower panel) of each taxon detected in the fecal samples of *Platalea minor* were shown. Colored bars represent 15 metazoan categories (at genus or higher taxonomic levels) based on 18S rDNA (For taxa categorization, see Supplementary Tables S1 and S3) and 29 fish species detected (at species or higher taxonomic levels).

We detected Actinopterygii in all samples (100% FOO) and frequently identified Decapoda in samples from GW10-MP and LMC-Jan (94.9% and 87.5% FOO; Figs. 3a and 4a; Supplementary Table S4; refer to the 'Study Site' in Methods and Materials for abbreviations). The proportion of Actinopterygii (63.9–98.1%% RRA) outweighed other consumed taxa, but high abundance of Decapoda was also detected in the samples of LMC-Jan (mean RRA = 22.3%), followed by GW10-MP (mean RRA = 17.1%). Samples collected in LMC-Mar contained a much higher abundance of Platyhelminthes (mean RRA = 31.4%) than samples from other roosting groups (0.6–2.9% RRA; Figs. 3a and 4a; Supplementary Table S5a). All detected orders of fish in this study were present in the GW10-MP group (Fig. 3b and 4b; Supplementary Table S6). Mullets accounted for 68.7% of all reads in GW10-MP samples, followed by tilapia *Oreochromis* spp. (16.5% RRA), eight species of gobies (total 12.1% RRA), and nine other minor fish species (0.01–1.08% RRA; Fig. 3b; Supplementary Table S7). Although tilapia *Oreochromis* spp. were the most abundant fish species in samples from GW21-MP and LMC-Mar (67.9% and 85.5% RRA; Fig. 3b; Supplementary Table S7), goby *R. giurinus* was the most common and abundant species in samples obtained in LMC-Jan (86.7% FOO and 65.1% RRA); Fig. 3b; Supplementary Tables S6 and S7).

While there was no obvious difference in the occurrences of identified taxa between individuals (Fig. 4a), a large variation in the relative abundance of each detected taxon existed between samples, particularly the RRA of Actinopterygii and Decapoda (0.91–100% RRA and 0–91.8% RRA, respectively; Fig. 4a). Large variations in the occurrence and relative abundance of each detected fish taxon also existed among individuals within the roosting groups (Fig. 4b). In 54 samples of GW10-MP, 40 mainly comprised *M. cephalus* and *Liza* spp. (60.2–100% RRA), nine were dominated by *Oreochromis* spp. (50.4–97.1% RRA), and the remaining samples mainly comprised crucian carps *Carassius* spp., gobies *P. taijiangensis*, or *G. giuris* (max. 56.8%, 56% and, 59.3% RRA, respectively; Fig. 4b). Six of eight GW21-MP samples were mainly composed of *Oreochromis* spp. (74.9–99.7% RRA), the other two mainly comprised *Coptodon* spp. and *Liza* spp. In LMC-Jan, 11 of 15 samples were dominated by *R. giurinus* (61.4–100% RAA), and the remaining were dominated by *Oreochromis* species. *Oreochromis* spp. was also dominant in most samples (n = 17 in 19) of LMC-Mar (57–100% RRA; Fig. 4b).

### Spatiotemporal variation in fecal taxa composition

**Figure 5:**
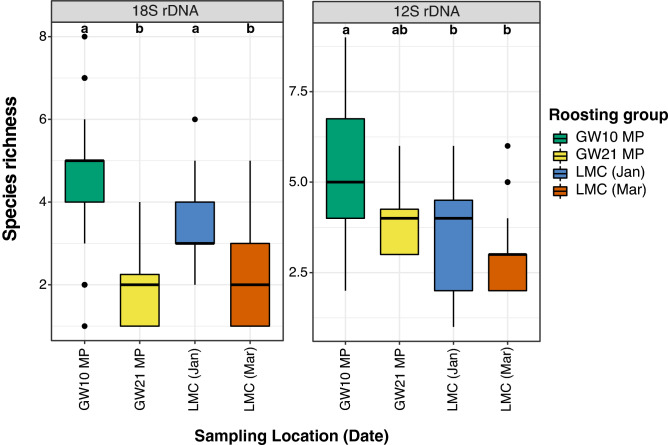
Incidence-based species richness of the dietary taxa in *Platalea minor* based on the 18S and 12S rDNA dataset (n = 110 and 96 respectively). The box represents the interquartile range. The line inside the box indicates the median. The upper whiskers extend to the highest value within 1.5 × the interquartile range, and the lower whiskers extend to the lowest value within 1.5 × the interquartile range. Black dots are outliers. Letters at the top of the boxes indicate significantly different groups. See Fig. 3 for abbreviations. The abundance-based figures and related tables are available in the Supplementary Fig. S1 and Table S8b.

The incidence-based species richness of fecal taxa composition (excluding Platyhelminthes) of GW10-MP detected using 18S rDNA (species richness = 4.6) were significantly higher than all other roosting groups except LMC-Jan (GW21-MP species richness = 2; LMC-Mar species richness=2.4, *p* < 0.05; Fig. 5 and Supplementary Table S8a). A significantly higher fish richness was also observed in GW10-MP using 12S rDNA (species richness = 5.1) compared to those of the LMC groups (species richness = 3–3.3, *p* < 0.05; Figs. 3b, 4b, and 5; Supplementary Table S8a).

**Figure 6:**
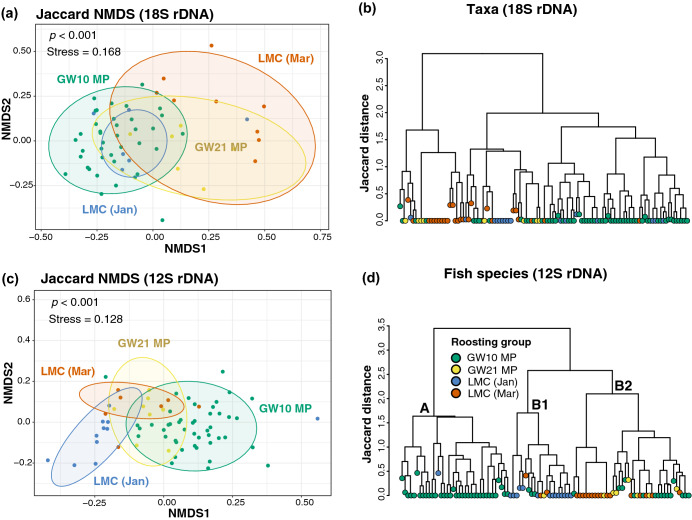
Incidence-based Jaccard dissimilarities of the dietary taxa compositions between different roosting groups of *Platalea minor*. **(a,c)** Nonmetric multidimensional scaling (NMDS) and **(b,d)** hierarchical clustering dendrograms showed the differences in dietary compositions of taxa **(a,b)** and fish species **(c,d)** between the roosting groups identified using 18S and 12S rDNA markers, respectively. Stress level of NMDS analysis and statistically significant differences examined by permutational ANOVA test (*p* < 0.001) were indicated. See Fig. 3 for abbreviations. The abundance-based figures and related tables are available in the Supplementary Fig. S2, Tables S5b, S7, S9b, and S10b.

Based on the Jaccard non-metric multidimensional scaling (NMDS), Ward’s hierarchical clustering and Pairwise PERMANOVA analysis of 18S and 12S rDNA, the fecal taxa compositions (including fish) were significantly different between samples from various roosting groups with some exceptions. For example, fecal taxa compositions identified by 18S rDNA were not significantly different between samples in GW21-MP and LMC-Mar (pairwise PERMANOVA, *p* = 1; Fig. 6; Supplementary Table S9a). However, these differences might be contributed by both intra- and inter-group variations (beta-dispersion: 18S, *p* = 0.0069; 12S, *p* = 0.0088).

Most samples in GW10-MP and LMC-Mar were significantly distinct in dietary compositions. The results of SIMPER analysis indicated that frequently detected mullets (*M. cephalus* and *Liza* spp., 34% of 12S variance), Gobiidae (*P. taijiangensis* and *H. hoevenii,* 13.8% of 12S variance), Decapoda (including Atyidae and *Penaeus* spp., 40.4% of 18S variance) and Haplotaxida (8.5% of 18S variance) in GW10-MP, as well as *Oreochromis* spp. in LMC-Mar (23.8% of 12S variance) contributed to the most of their dissimilarity (Figs. 4 and 6; Supplementary Tables S4, S6, S9a, and S10a).

Samples in LMC-Jan shared similar 18S taxa compositions with samples in GW10-MP and LMC-Mar (Fig. 6a,b), and yet the fish species present in these groups were largely different from each other (Fig. 6c,d). The highly prevalent *Coptodon* spp., *R. giurinus*, *P. parva*, *G. affinis* in LMC-Jan (1.5–19.6% of 12S variance), *Oreochromis* spp. (29.3% of 12S variance) in LMC-Mar, as well as mullets (*M. cephalus* and *Liza* spp., 31.8% of 12S variance) and *P. taijiangensis* (7.8% of 12S variance) in GW10-MP were mainly responsible for these variations (Figs. 4 and 6; Supplementary Tables S6 and S10a). Diversity of dietary taxa of different roosting groups based on read abundance data, which shows similar results, were provided in the Supplemental Information (Supplementary Fig. S1, S2, Tables S8b, S9b, and S10b).

## Discussion

### Dietary composition of *P. minor*

We identified a high diversity and variation in the diets of wintering *P. minor*. A substantial proportion of their diets was species in the orders Mugiliformes and Cichliformes, which is consistent with the high abundance of these taxa found in the stomachs of *P. minor* carcasses during winter in Taiwan^28^. However, apparent differences existed in the compositions and abundances of the detected species between the two studies. For instance, *Yongeichthys caninus* (aka *Acentrogobius caninus*, tropical sand goby, Gobiiformes) frequently observed in the diets of *P. minor* wintering in Taiwan was absent in our study, while *M. cephalus* was only present in low abundance in samples from Taiwan. Some prey consumed by *Platalea* spp. in other places, such as flatfish and toadfish, were not detected in our study^29^. Since *P. minor* is purported to be an opportunistic feeder^21^, the variations in diets observed could be due to the differences in aquatic faunal composition between foraging sites.

Although *P. minor* is highly adapted to foraging in shallow coastal waters, their feeding strategy enables them to eat a wide variety of aquatic prey, and the species could, therefore, be considered a generalist. Interestingly, some fish species occurring in considerable abundance in the Deep Bay were barely detectable or completely absent in the *P. minor* diet identified in this study. Examples include *A. gymnocephalus* (bald glassy, Perciformes), *Boleophthalmus pectinirostris* (great blue spotted mudskipper, Gobiiformes), and *Periophthalmus cantonensis* (common mudskipper, Gobiiformes)^30–32^, and all these small fishes are similar to the common prey of *P. minor* in being euryhaline and either demersal or benthopelagic^33^. Considering *P. minor* is an opportunistic tactile feeder, the reason why the birds did not consume these abundant species is not clear. One possible explanation is that they have a certain degree of food preference. The idea of food preference was also reported for *P. leucorodia* in south-western Spain. In Spain, they ate prawns in a relatively smaller proportion than expected based on the population density of prawns at the foraging site^34^, and the size or profitability of prey was suggested to be a determinant. An alternative explanation is a difference in the mobility of different prey species, such as swimming speed, response time, and reaction to environmental stimuli, which may mean that some prey is easier for *P. minor* to capture than others. The possible prey preference of this endangered species as a whole or at the individual level, or the possibility that some prey species are easier to catch than others, will have conservation implications, especially for the design and maintenance of artificial wetlands.

### Dietary composition variation between roosting groups

Ours is the first study to clearly demonstrate a clear pattern of spatiotemporal variations in dietary composition between roosting groups and individuals of the endangered *P. minor* through the high taxonomic resolution of DNA metabarcoding. Our results showed that the dietary composition of individuals within the same roosting group was more similar than that between the groups, and such similarities were observed in all roosting groups. Despite the possibility that some of the fecal samples collected on different sampling dates in the same site might belong to the same individuals, our findings strongly indicate that individuals roosting at the same location commonly forage in the same habitat or habitats with very similar prey composition on the same day. For example, most individuals in GW21-MP almost entirely fed on Actinopterygii, with *Oreochromis* spp. being the major component in their diets. The dietary compositions of the roosting groups differed considerably from each other. For example, the roosting group GW10-MP mainly fed on Actinopterygii and Decapoda, and ate a very high diversity of fish prey, with Mugiliformes being the most frequently consumed. In contrast, the group from LMC-Mar almost exclusively consumed fish and barely any crustaceans, with fish in the Cichliformes being the most frequently eaten. Diversity analysis of 12S rDNA suggested that samples from LMC presented significantly lower dietary diversity than those from GW10-MP. In addition, temporal variations existed between groups in the same sampling location. For instance, groups from LMC-Jan consumed more decapods than LMC-Mar. Moreover, we did not detect gobies at all in the diets of most individuals from LMC-Mar, while gobies made up the majority of the LMC-Jan diet. This variation suggests that the group(s) of birds roosting around the same location at different time points might use different habitats for foraging during the wintering period. Alternatively, more gobies might be available in January in the areas they foraged.

A previous study has documented observational data on *P. minor* feeding in different habitat types during winter in Asian regions, including intertidal mudflats in sheltered estuaries, fishponds, and gei wai, all within 2 to 3 km of tidal water^35^. Considering *P. minor* is an opportunistic feeder, the pronounced pattern in dietary composition between groups might reflect similar behaviors; groups with different diets foraged in different habitat, which have different aquatic faunal compositions. For instances, the shared characteristic between the diets of GW21-MP and LMC-Mar was their predominant consumption of tilapia, which indicates that these two groups might both forage in habitats such as the mitigation ponds stocked with tilapia or some estuarine microhabitats that have naturally occurring tilapia. The dietary composition of LMC-Jan has shown that the birds fed a lot more on *R. giurinus* in the Gobiiformes, which was almost absent from the diets of other groups. Cichliformes and Decapoda accounted for a portion of LMC-Jan. Gobiiformes, such as *R. giurinus*, are known to exist abundantly in the natural shallow intertidal mudflats^33^, and Cichliformes and Decapoda also naturally occur in the bay^36^. Based on the species diversity and composition, it is very likely that individuals in LMC-Jan foraged at the intertidal mudflats.

### Dietary composition variation between individuals within a roosting group

Our results also revealed a considerable variation in diets within the same roosting group; indeed, such intraspecific variations were observed in all roosting groups. For example, despite the taxa in the diets of individuals from GW10-MP being similar, the relative abundance of each of these detected taxa was highly variable between individuals. In GW10-MP, *M. cephalus* was detected in a wide range of relative abundances, ranging from approximately 0.2% to 93% among the samples. Similarly, individuals from LMC-Jan also consumed variable amounts of decapods, *R. giurinus* and *Oreochromis* spp. These intraspecific dietary variations within the same group may be attributed to several factors. If individuals from the same group foraged at the same site, individual preference for prey of particular taxa or species could be a plausible explanation. Given the certain degree of food preference exhibited by *P. minor*, individual preferences may also occur, as discussed earlier. For instance, individuals could selectively forage for some prey but reject others because of lack of the palatability^37^. Individual preferences might also be associated with different life-history traits of an individual, such as sex and age. Differences in food preferences between juveniles and adults have been reported in birds, such as oystercatchers (*Haematopus* spp.) and little bustards (*Tetrax tetra*)^38,39^. Significant sex-dependent food preference or intersexual plasticity in the diet has been observed in different animals, such as storm petrels, oystercatchers, grouses, crows, minks, bats, and capuchins^40–46^. An alternative explanation for individual dietary variation is the foraging proficiency of individuals. Individuals might possess a series of inherited or learned predatory abilities to catch nekton at specific sizes or swimming speeds^47,48^. Bill length, body condition, or experience could affect the successful rates of capturing different prey. In addition, the observed variations could simply be due to the prey encountered in the microhabitat in which each individual foraged. Instead of variations in prey preferences or hunting abilities, they might just consume any prey they could catch, reflecting the variations due to random sampling. Lastly, even though most individuals in the same roosting group had similar dietary compositions, some individuals might have foraged in different sites or habitats.

### Conservation implications

Food availability is one of the most crucial factors affecting the survival of waterbirds in winter, which is the toughest season for wildlife. The current study demonstrates that the endangered *P. minor* is an opportunistic generalist, which is predominantly piscivorous and exhibits dietary diversity during the wintering period. We found that *P. minor* consumed at least 26 species in the classes Actinopterygii and Malacostraca, as well as many other aquatic organisms in smaller proportions. Mugiliformes, Cichliformes and Gobiiformes are some of the key taxa that contributed most to their diets. The detailed dietary spectra identified in this study reveal the composition of prey in the wetland habitats that can be critical in refueling and nourishing the birds during migration, and sustaining the global wild population in the long term. Conservation measures must be taken to protect the existence and abundance of these essential food sources. Using the high taxonomic resolution of DNA metabarcoding, this study also presents evidence on the spatiotemporal and intraspecific variations in the diets of this species for the first time, potentially reflecting some of their feeding habits. Roosting groups appear to forage in different habitat types during the winter and some individuals in the same roosting group tend to forage in flocks based on their diets. This information suggests that these different habitats under proper management provide important ecological functions and they are vital for harboring the suitable food sources for waterbirds. More importantly, our study demonstrates the applicability of combining high-throughput sequencing techniques and non-invasive sampling for studying the diets of threatened species, and this powerful tool can provide invaluable knowledge important for species conservation.

## Materials and methods

### Study site

We collected samples at the north-western part of Hong Kong, which contains a wetland complex (22°29′48.5" N 114°02′02.5" E) comprising natural habitats of mudflats and mangroves on the eastern bank of the Pearl River estuary of Deep Bay, and a large area of semi-natural gei wai and artificial fishponds bordering the southern part of Inner Deep Bay and the land of Shenzhen (Fig. 1)^13^. Previous studies have reported that several fish taxa dominate the intertidal mudflat, including gobies, mullets, tilapia, and mosquitofish^36^. Traditional drain-down practices are maintained in the semi-natural habitat, gei wai, in the Mai Po Marshes to rear shrimps and fish (without feeding) for wintering waterbirds as a conservation measure^36^. The adjacent large area of artificial fishponds is the predominant habitat type in the wetland complex. Many commercial fishponds are rain-fed ponds used for polyculture of mullets, carps (including goldfish *Carassius auratus*, grass carp *Ctenopharyngodon idella*, bighead carp *Hypophthalmichthys nobilis*, mud carp *Cirrhinus molitorella*, common carp *Cyprinus carpio*, freshwater bream *Abramis brama*, and silver carp *Hypophthalmichthys molitrix* in the family Cyprinidae) and tilapia, with total annual fish fry stocking of 3,972,600, 1,439,650 and 152,100 tails, respectively, in 2018–2019 (unpublished data, AFCD). Conservation measures are also being undertaken at the mitigation ponds of Lok Ma Chau (LMC) to compensate for wetland loss due to the construction of the LMC Spur Line. One measure was repeated stocking of ponds with fish of little economic value, mainly tilapia, during winter months^49^. In addition, more than 600 ha of the aquaculture area is drained or partially drained during winter months for commercial harvesting and simultaneously aims to provide food for migratory waterbirds as a conservation initiative implemented by the Hong Kong Government’s Management Agreement Programmes^50^.

We collected fecal samples from three sites where *P. minor* commonly roosted in the Deep Bay wetland. Two sampling sites were located at the gei wai (Fig. 1), which were Gei Wai 10 (GW10-MP; 22°29′34.994" N 114°2′24.716" E) and Gei Wai 21 (GW21-MP; 22°29′0.589" N 114°2′3.832" E)^36^. These sampling sites are mainly fringed by intertidal mudflats, mangroves, other gei wai and fishponds. The remaining sampling site was at a mitigation fishpond inside the LMC ecological enhancement area (22°30′41.663" N 114°3′55.645" E). It is surrounded by fishponds to the south and a highly developed city area of Shenzhen to the north. Permissions to enter sampling sites were obtained.

### Sample collection and DNA metabarcoding

During early mornings, we collected fresh feces of *P. minor* on the ground beside ponds from where the birds had defecated shortly after leaving the ponds. Live animals were not involved, and ethics approval was not required. Samples collected were spaced at least ~ 0.5 m apart to avoid repeat sampling of the same individual. We collected 111 fecal samples between January and March 2019 (Supplementary Table S11). Samples were preserved in absolute ethanol in the field and stored at − 80 °C later on the same day until DNA extraction. We used the QIAamp Fast DNA Stool Mini Kit (Qiagen, Hilden, Germany) to extract fecal DNA. We performed DNA barcoding on each sample and confirmed that 110 samples were from *P. minor* (Supplementary Materials and Methods). We included mock communities and negative controls during DNA extraction in our analyses (Supplementary Table S12). DNA quantifications was performed using Qubit dsDNA high-sensitivity (HS) assays on a Qubit 4 Fluorometer (Invitrogen**,** Carlsbad, CA).

As the major prey of *P. minor* included fishes and other animal taxa such as crustaceans^21^, we used a set of primers universal for metazoans (18s_SSU3_F: 5'GGTCTGTGATGCCCTTAGATG3' and 18s_SSU3_R: 5'GGTGTGTACAAAGGGCAGGG3') that amplifies the V7 region of 18S small subunit of nuclear ribosomal DNA (rDNA; ~ 170 bp) from all major animal lineages^25^. This V7 region has a taxonomic resolution at a higher taxonomic level and recognizes a broader spectrum of the diet. We also used another set of primers specific for fish mitochondrial 12S rDNA (MiFish-U-F: 5'GTCGGTAAAACTCGTGCCAGC3' and MiFish-U-R: 5'CATAGTGGGGTATCTAATCCCAGTTTG3'), which amplify a hypervariable region (~ 163 bp) to identify the majority of fishes to species taxonomically^51^. After removing low quality samples, 110 18S and 96 12S fecal DNA samples, mock communities, and negative controls were used for library preparation of 18S and 12S rDNA markers through two-step polymerase chain reaction (PCR) (Supplementary Materials and Methods). A library multiplex was created by pooling libraries from different samples together in an equimolar ratio. Each library was sequenced to a depth of approximately 400 k reads on a NovaSeq instrument (PE 150 bp reads) by Novogene Corporation (Beijing, China).

### Bioinformatics

We pre-processed demultiplexed raw 18S and 12S paired-end fastq reads by paired read merging, adapter trimming, and quality filtering. Paired-end reads were merged using USEARCH v11.0.667^52^ with the -fastq_mergepairs function. PCR primer sequences were trimmed using CUTADAPT v2.4^53^ with the *linked adapter* mode (max_error_rate = 0.1). We retained only the merged reads with matched 12S or 18S primer sequences. The quality of reads was assessed using FastQC v0.11.8^54^ and the -fastq_eestats2 command of VSEARCH^55^. Then, we retained high-quality reads within the target length (18S, 130–220 bp; 12S, 130–260 bp) and with expected errors per read less than one (-fastq_maxee 1) using the -fastq_filter function in VSEARCH. All pre-processed reads were dereplicated (VSEARCH -derep_fulllength), from which the zero-radius operational taxonomic units (ZOTUs) or amplicon sequence variants (ASVs) were generated by removing chimeras and singletons (with abundance less than 20 reads) using USEARCH -unoise3^56,57^. We clustered all pre-processed reads into ASVs using a 99% similarity threshold (VSEARCH -usearch_global -id 0.99).

We assigned the taxonomy of each ASV to the lowest identifiable taxonomic level using the SINTAX algorithm in USEARCH^58^ or assigned a taxonomy function in R package DADA2 v1.12.1^59^ with a bootstrap cutoff of 0.7. The mitochondrial reference databases MIDORI^60^ and MitoFish^61^ were used for 12S rDNA dataset. The ribosomal RNA database SILVA (release 132, July 2017)^62^ and the NCBI nt database (non-redundant nucleotide sequences) were used for 18S rDNA dataset. We assigned the lowest common taxonomic level shared by all 18S rDNA blast hits with an E-value less than 1e-50 and similarity above 99% using BASTA^63^ with the algorithm of the lowest common ancestor (LCA). Non-dietary ASVs and contaminant ASVs determined by negative controls, e.g., *Homo sapiens*, *Platalea* spp., and *Tiarina* spp., were discarded. Potential false positives in the samples were filtered out by applying thresholds defined by mock communities (see details in Supplementary Materials and Methods, Supplementary Tables S13 and S14).

### Data analysis

We identified 551 ASVs and 183 taxa for 18S, and 40 ASVs and 29 taxa for 12S after filtering (Supplementary Materials and Methods, Supplementary Tables S15 and S16). While the lowest identifiable taxonomic level in 18S taxa ranged from kingdom to genus (1 kingdom, 28 phyla, 63 classes, 76 orders, 58 families, and 81 genera; Supplementary Table S15), the taxonomy of most 12S taxa could be resolved down to the species level (1 phylum, 7 orders, 9 families, 21 genera and 21 species; Supplementary Table S16). For the 18S dataset, apart from taxa in the class Actinopterygii and Malacostraca, we grouped all other taxa into 16 larger categories based on the taxonomic information, in which 7 non-metazoan categories, including Eukaryota, Fungi, Algae, Protozoa, Plant, Fungus-like, and Fornicata were further removed, leaving 40 taxa for downstream analyses (Supplementary Table S15). We summarized the filtered data as (i) percentage of read count for each taxon in a sample (relative read abundance, RRA), (ii) percentage of occurrence for each taxon in a sample (weighted percentage of occurrence, wPOO) and (iii) proportion of samples in which a taxon was detected (frequency of occurrence, FOO)^64^ (Supplementary Tables S17-S18).

After removing Platyhelminthes, the non-dietary category, we compared the diversity of dietary taxa within and between roosting groups by using incidence-based metrics, which are not subject to biases in read abundance resulted from factors such as differences in prey size, prey digestibility, PCR biases, etc. Only the presence and absence of each taxon in samples within each roosting group was used in incidence-based diversity analyses (see Supplemental Information for abundance-based analyses). For alpha diversity analysis, species richness in each sample was evaluated by using direct count of taxa. The species richness of roosting groups was plotted using ggplot2. We used Tukey's honest significant difference (Tukey’s HSD) analysis to estimate the pairwise differences in diversity indices between roosting groups.

We inferred differences in dietary taxa composition in samples among roosting groups by calculating the pairwise Jaccard dissimilarity distance based on incidence of each taxon. We visualized the results with hierarchical clustering analysis using Ward’s method in hclust function and NMDS ordination using the metaMDS function (*k* = 2) in vegan v2.5-6^66^ and phyloseq v1.28.0^67^ in R. Permutational ANOVA (PERMANOVA) test was utilized to evaluate the statistical significance of the inter-group variations of taxa composition, which has the prerequisite for homogeneity of intra-group beta-dispersion (*p* > 0.05). We tested pairwise PERMANOVA of roosting groups using pairwise adonis^68^ and evaluated their beta-dispersion with the betadisper function in vegan. We tested the contribution of individual taxon to the variations between roosting groups using similarity percentage (SIMPER) based on the Jaccard dissimilarities, and their statistical significance was evaluated using the non-parametric Kruskal–Wallis rank-sum test. These were carried out using functions simper.pretty and kruskal.pretty in R scripts simper_pretty.R and R_krusk.R. Only taxa that contributing more than 1% of the variance (*p* < 0.05) were presented^69^.

## Supplementary Information

Supplementary Information.

## Data Availability

Raw 18S rDNA and 12S rDNA sequences and sample information in this study are available in the NCBI Sequence Read Archive (SRA) under BioProject accession PRJNA758792. Biological information for samples, mock communities and negative controls are available under BioSample accessions SAMN21165410-5615, SAMN21192046-2055, and SAMN21192196-2199, respectively. Raw sequences are available under SRA accession SRP335461.
