# Supplementary material for: DNA metabarcoding reveals the dietary composition in the endangered black-faced spoonbill
Source: Sci Rep. 2021 Sep 21;11:18773. doi: 10.1038/s41598-021-97337-w (PMC8455529; doi:10.1038/s41598-021-97337-w)
Supplement: Supplementary file 1 — Supplementary Information. [file 41598_2021_97337_MOESM1_ESM.pdf]

**Supplemental Information for:**

# DNA metabarcoding reveals the dietary composition in the endangered black-faced spoonbill

Pei-Yu Huang [a,d], Emily Shui Kei Poon [a,d], Anson Tsz Chun Wong [a], Ivy Wai Yan So [b], Yik Hei Sung [c], Simon Yung Wa Sin [a*]

**Author affiliation**

[a] School of Biological Sciences, The University of Hong Kong, Pok Fu Lam Road, Hong Kong SAR.

[b] Wetland and Fauna Conservation Division, Agriculture, Fisheries and Conservation Department, Hong Kong SAR Government, Hong Kong SAR.

[c] Science Unit, Lingnan University, Tuen Mun, Hong Kong SAR.

[d] These authors contributed equally.

*** Corresponding author**

Simon Yung Wa Sin

Email: yungwa.sin@gmail.com

Supplementary materials and methods

Tables S1-S18

Figures S1-S2

## Supplementary Materials and Methods

### *DNA barcoding to identify host species*

We designed a set of primers to specifically amplify a DNA minibarcode (116 bp) from the mitochondrial cytochrome b (cytb) gene of *Platalea* spp., by aligning the cytb sequences from *Platalea* species, including *P. minor*, and other waterbird species that commonly winter in Hong Kong. PCRs were performed in 20 µl reactions comprising 4 µl of 5X GoTaq Flexi Buffer, 0.4 µl of 10 mM dNTP Mix, 2.4 µl of 25 mM $MgCl_2$, 0.2 µl of 5 U/µl GoTaq G2 Flexi DNA Polymerase (all from Promega), 5 µl of extracted DNA, 0.4 µl of 10 µM forward primer Cyb_657F (5'CCCATTCCACCCCTACTTTTCA3'), 0.4 µl of 10 µM reverse primer Cyb_772R (5'TGAAGTTTTCGGGGTCACCT3'), 2 µl of 10 % dimethyl sulfoxide (DMSO) (Sigma), 0.1 µl of 20 mg/ml bovine serum albumin (BSA) (New England Biolabs) and 5.1 µl of UltraPure DNase/RNase-Free Distilled Water (ultrapure water) (Invitrogen, Carlsbad, CA). Touchdown PCRs were used with the following condition: 95 °C for 2 min; 10 cycles of 95 °C for 30 sec, 65-55 °C for 30 sec with 1 °C decrease per cycle and 72 °C for 30 sec; 25 cycles of 95 °C for 30 sec, 53 °C for 30 sec, and 72 °C for 30 sec; and final extension at 72 °C for 5 min. All DNA minibarcodes obtained were sanger sequenced to confirm the identities of host species. Sequencing was carried out by the BGI (Shenzhen, China).

### *Preparation of mock communities*

We obtained eight specimens of aquatic species, including six species in class Actinopterygii, one species in class Malacostraca, and one species in class Gastropoda, from local food market (Supplementary Table S12). We used the E.Z.N.A. Tissue DNA Kit (Omega Bio-tek, USA) to extract gDNA from the fresh tissues of these species. Five mock communities (MC0-MC4) were prepared according to the Supplementary Table S12, which shows the species compositions of each mock community. MC0 was prepared by mixing 5mg (wet weight) of tissue from each of the included species for DNA extraction, whilst MC1-4 were prepared by mixing 15ng of gDNA from each of the included species. The number of species included in each of these mock communities ranged from five to eight, these numbers of species were in a similar range to the numbers of prey species detected in individual *P. minor* samples.

### *Preparation of libraries through 2-step PCRs*

For 18S libraries, 1$^{st}$ step PCRs were carried out in 25 µl reactions containing 5 µl of 5X GoTaq Flexi Buffer, 0.5 µl of 10 mM dNTP Mix, 3 µl of 25 mM $MgCl_2$, 0.25 µl of 5 U/µl GoTaq G2 Flexi DNA Polymerase, 5 ng of extracted DNA, 0.5 µl of each assigned 10 µM forward and reverse primer uniquely tagged with heterogeneity spacer (Cruaud et al. 2017), 5 µl of 10 % DMSO (Sigma), 0.125 µl of 20 mg/ml BSA (NEB) and ultrapure water. Thermal cycling condition was 95 °C for 2 min; 25 cycles of 95 °C for 30 sec, 65 °C for 30 sec and 72 °C for 30 sec; and final extension at 72 °C for 5 min. We used the PureLink PCR Purification Kit (Invitrogen, Carlsbad, CA) to clean up all PCR products generated from the two-step PCRs. Each of the 1$^{st}$ step PCR products was eluted in 20 µl elution buffer and used as the templates for the 2$^{nd}$ step PCRs. 2$^{nd}$ step PCRs were performed in 45 µl reactions comprising 9 µl of 5X GoTaq Flexi Buffer, 0.9 µl of 10 mM dNTP Mix, 5.4 µl of 25 mM $MgCl_2$, 0.225 µl of 5 U/µl

GoTaq G2 Flexi DNA Polymerase, 20 µl of 1st step PCR products, 0.9 µl of each assigned 10 µM forward and reverse primer tagged with Illumina adapter and unique multiplexing index (Cruaud et al. 2017), 4.5 µl of 10 % DMSO (Sigma) and ultrapure water. PCR condition was 95 °C for 2 min; 10 cycles of 95 °C for 30 sec, 55 °C for 30 sec and 72 °C for 30 sec; and final extension at 72 °C for 5 min. For 12S libraries, we used a high fidelity polymerase for the 2-step PCRs. The 1st step PCRs were carried out in 25 µl reactions comprising 5 µl of 5X Phusion HF Buffer, 0.125 µl of 2 U/µl Phusion Hot Start Flex DNA Polymerase, 0.125 µl of 20 mg/ml BSA (all from NEB), 0.5 µl of 10 mM dNTP Mix (Promega), 5 ng of extracted DNA, 0.5 µl of each assigned 10 µM forward and reverse primer uniquely tagged with heterogeneity spacer, 5 µl of 10 % DMSO (Sigma) and ultrapure water. Thermal cycling condition was 98 °C for 2 min; 25 cycles of 98 °C for 30 sec, 53 °C for 30 sec and 72 °C for 30 sec; and final extension at 72 °C for 5 min. The 2nd step PCRs were performed in 45 µl reactions containing 9 µl of 5X Phusion HF Buffer, 0.225 µl of 2 U/µl Phusion Hot Start Flex DNA Polymerase (all from NEB), 0.9 µl of 10 mM dNTP Mix (Promega), 20 µl of 1st step PCR products, 0.9 µl of each assigned 10 µM forward and reverse primer tagged with Illumina adapter and unique multiplexing index (Cruaud et al. 2017), 4.5 µl of 10 % DMSO (Sigma) and ultrapure water. PCR condition was 98 °C for 2 min; 10 cycles of 98 °C for 30 sec, 55 °C for 30 sec and 72 °C for 30 sec; and final extension at 72 °C for 5 min.

***Data pre-processing and filtering***

We used negative controls to eliminate potential contaminant ASVs and mock communities to determine the thresholds for removing false positive reads. To make the read count comparable between samples and negative controls, total read count of each ASV from all samples was normalized by dividing it by its fold change compared to that of all negative controls. ASVs with read count in negative controls an order of a magnitude more than that in normalized sample read count were regarded as contaminants and discarded (Lee et al. 2015). All of the false-positives in mock communities of 18S and 12S libraries could be eliminated by applying threshold levels of 0.08% and 0.02%, respectively (Supplementary Tables S13 and S14). To ensure the removal of false-positives in all samples, we applied a more stringent threshold levels of 0.1% and 0.05% to 18S and 12S, respectively. Low threshold should be applied only when the requirement of high sequencing depth for each sample is fulfilled (>10,000 reads per sample) (Deagle et al. 2019). In this study, the lowest read count per sample was 49,651 and the low threshold used here is justifiable.

Sequencing of samples, mock communities and negative controls yielded quality-filtered paired-end raw reads 144,148,356 from 111 samples of 18S and 99,341,650 from 97 samples of 12S. After merging the paired-end sequences, we retained 70,608,252 and 48,311,373 reads for 18S and 12S, respectively. Followed by primer trimming, quality filtering and removal of chimera and singletons, 58,129,311 and 44,057,306 reads, accounted for 82% and 91% of total merged reads for 18S and 12S, respectively, were retained (mean number of reads per sample: 457,272 and 382,269 for 18S and 12S loci, respectively after pre-processing). The sample from *Platalea leucorodia* (Eurasian spoonbill) was excluded from later analyses. We removed false-positives with thresholds based on mock communities and removed contaminant ASVs based on negative controls. After removal of false-positives and contaminants, we retained 553 from 933 ASVs and 184 from 233 taxa for 18S. For 12S rDNA, we kept 43 from 55 ASVs and 31 from 38 taxa for later analyses. Then, we excluded reads from non-target taxa, such as *Homo sapiens* and *Platalea* spp., leaving 551 ASVs and 183 taxa for 18S, and 40 ASVs and 29 taxa

for 12S (Tables S15 and S16). The 143 non-metazoan 18S taxa in 7 categories including Eukaryota, Fungi, Algae, Protozoa, Plant, Fungus-like, and Fornicata were removed, leaving 40 taxa in the analyses (Fig. 2-4). Non-dietary category Platyhelminthes was excluded from the dietary diversity analysis of 18S (Fig. 5-6). Aves (*Platalea* spp.) accounted for 14.0% and 29.3% of the sample reads for 18S and 12S (Supplementary Tables S17 and S18).

***Diversity analyses of abundance-based data***

While incidence-based data only takes into account the presence and absence of each taxon, abundance-based data includes the information of read abundance of each taxon. The alpha diversity of abundance-based dietary taxa of roosting groups was estimated by Shannon's diversity index (H), which takes into account both species abundance and evenness, and Simpson's dominance index (D), which determines whether the community is dominated by a few taxa by R package microbiome v1.6.0 (Lahti and Shetty 2017) and plotted with ggplot2. We used Tukey Honest Significant Difference (Tukey's HSD) analysis to estimate the pairwise differences of diversity indices between roosting groups.

We inferred differences of dietary taxa composition in samples among roosting groups by calculating the pairwise Bray-Curtis dissimilarity distance based on fourth root transformed relative read abundance of each taxon. Please refer to Materials and Methods in the main text for the non-metric multidimensional scaling (NMDS), Ward's hierarchical clustering, and Pairwise PERMANOVA analyses.

**References**


Cruaud P, Rasplus J-Y, Rodriguez LJ, Cruaud A (2017) High-throughput sequencing of multiple amplicons for barcoding and integrative taxonomy Scientific reports 7:41948

Deagle BE et al. (2019) Counting with DNA in metabarcoding studies: How should we convert sequence reads to dietary data? Molecular Ecology 28:391-406 doi:10.1111/mec.14734

Lahti L, Shetty S (2017) Tools for microbiome analysis in R. Microbiome package version. http://microbiome.github.io/microbiome.

Lee MD, Walworth NG, Sylvan JB, Edwards KJ, Orcutt BN (2015) Microbial communities on seafloor basalts at Dorado Outcrop reflect level of alteration and highlight global lithic clades Frontiers in microbiology 6:1470

**Table S1** Taxa composition identified by 18S fecal rDNA of *Platalea minor* after removal of non-metazoan taxa. Abbreviations: RRA, relative read abundance; wPOO, weighted percentage of occurrence; FOO, frequency of occurrence.

| Taxa | % RRA | % wPOO | % FOO | No. of Taxa |
|---|---|---|---|---|
| **Actinopterygii** | **74.55** | **35.95** | **100** | **4** |
| **Malacostraca** | **15.20** | **33.20** | **73.64** | **5** |
| Malacostraca (Class) | 0.01 | 0.89 | 3.64 | 1 |
| Decapoda (Order) | 12.78 | 20.89 | 72.73 | 1 |
| Atyidae (Family) | 0.34 | 5.63 | 24.55 | 1 |
| *Penaeus* spp. | 2.08 | 5.61 | 25.45 | 1 |
| *Neomysis* spp. | 0.00 | 0.18 | 0.91 | 1 |
| **Vertebrata** | **0.11** | **5.08** | **20.91** | **1** |
| **Platyhelminthes** | **8.50** | **11.41** | **40.91** | **11** |
| **Zooplankton** | **0.21** | **3.73** | **18.18** | **5** |
| **Annelida** | **0.75** | **5.56** | **23.64** | **2** |
| **Sponge** | **0.19** | **1.27** | **4.55** | **3** |
| **Arachnid** | **0.19** | **0.71** | **2.73** | **1** |
| **Insecta** | **0.02** | **0.98** | **2.73** | **2** |
| **Cnidaria** | **0.00** | **0.33** | **1.82** | **2** |
| **Nematoda** | **0.26** | **1.77** | **8.18** | **4** |
| **Total** | **100** | **100** | **-** | **40** |

**Table S2** Read abundance, occurrence and taxonomy of Platyhelminthes detected by 18S rDNA.

| Taxonomic ranks | Read abundances and occurrences | | | | Taxonomies | | | | | |
|---|---|---|---|---|---|---|---|---|---|---|
| | Counts | %RRA | %wPOO | %FOO | Kingdom | Phyla | Classes | Orders | Families | Genera |
| **Cyclophyllidea** | 1.6755E+13 | 28.95 | 24.44 | 33.33 | Eukaryota | Platyhelminthes | Cestoda | Cyclophyllidea | | |
| ***Paradilepis* spp.** | 617845 | 13.53 | 12.96 | 24.44 | Eukaryota | Platyhelminthes | Cestoda | Cyclophyllidea | Gryporhynchidae | *Paradilepis* |
| **Diplostomida** | 237446 | 32.72 | 34.81 | 44.44 | Eukaryota | Platyhelminthes | Trematoda | Diplostomida | | |
| **Schistosomatidae** | 166275 | 8.71 | 7.04 | 11.11 | Eukaryota | Platyhelminthes | Trematoda | Diplostomida | Schistosomatidae | |
| **Stenostomidae** | 10395 | 7.23 | 8.89 | 13.33 | Eukaryota | Platyhelminthes | Catenulida | Catenulida | Stenostomidae | |
| **Platyhelminthes** | 4587 | 4.49 | 5.56 | 6.67 | Eukaryota | Platyhelminthes | | | | |
| **Mazocraeidea** | 1934 | 2.22 | 2.22 | 2.22 | Eukaryota | Platyhelminthes | Monogenea | Mazocraeidea | | |
| **Rhabdocoela** | 598 | 1.20 | 1.11 | 2.22 | Eukaryota | Platyhelminthes | Rhabditophora | Rhabdocoela | | |
| **Monogenea** | 589 | 0.72 | 1.11 | 2.22 | Eukaryota | Platyhelminthes | Monogenea | | | |
| **Heterophyidae** | 1327 | 0.13 | 0.74 | 2.22 | Eukaryota | Platyhelminthes | Trematoda | Opisthorchiida | Heterophyidae | |
| **Cestoda** | 424 | 0.11 | 1.11 | 2.22 | Eukaryota | Platyhelminthes | Cestoda | | | |

**Table S3** Fish species composition identified by 12S fecal rDNA of *Platalea minor* after removal of non-dietary taxa. Abbreviations: RRA, relative read abundance; wPOO, weighted percentage of occurrence; FOO, frequency of occurrence.

| Orders | Genera/Species | Common names | % RRA | % wPOO | % FOO |
|---|---|---|---|---|---|
| **Mugiliformes** | ***Mugil cephalus*** | **Grey mullet** | **21.23** | **13.04** | **59.38** |
| | ***Liza* spp. (All)** | **-** | **21.35** | **20.47** | **60.82** |
| | *Liza* spp. | - | 15.23 | 12.57 | 56.25 |
| | *L. subviridis* | Greenback mullet | 3.84 | 6.27 | 32.29 |
| | *L. affinis* | Eastern keelback mullet | 2.274 | 1.64 | 8.33 |
| | ***Valamugil* spp.** | **-** | **0.02** | **0.73** | **4.17** |
| **Cichliformes** | ***Oreochromis* spp. (All)** | **Tilapia** | **34.45** | **29.77** | **65.98** |
| | *Oreochromis* spp. | - | 16.28 | 13.74 | 52.08 |
| | *O. niloticus* | Nile tilapia | 18.17 | 16.04 | 61.46 |
| | ***Coptodon* spp.** | **-** | **2.83** | **6.05** | **27.08** |
| **Gobiiformes** | ***Rhinogobius giurinus*** | **Barcheek goby** | **10.4** | **6.54** | **20.83** |
| | ***Pseudogobius taijiangensis*** | **Taijiang fat-nose goby** | **3.53** | **6.16** | **32.29** |
| | ***Glossogobius giuris*** | **Tank goby** | **2.46** | **2.84** | **10.42** |
| | ***Hemigobius hoevenii*** | **Banded mullet goby** | **0.69** | **2.32** | **14.58** |
| | ***Gobiopterus lacustris*** | **Lacustrine goby** | **0.41** | **1.55** | **7.29** |
| | ***Mugilogobius* spp.** | **-** | **0.29** | **0.96** | **4.12** |
| | *M. abei* | Estuarine goby | 0.18 | 0.61 | 3.13 |
| | *M. chulae* | Yellowstripe goby | 0.11 | 0.35 | 1.04 |
| | ***Favonigobius gymnauchen*** | **Sharp-nosed sand goby** | **0.008** | **0.15** | **1.04** |
| | **Gobiiformes (Order)** | **-** | **0.35** | **0.56** | **3.13** |
| | **Gobiidae (Family)** | **-** | **0.005** | **0.17** | **1.04** |
| **Cypriniformes** | ***Carassius* spp.** | **Crucian carps** | **1.09** | **1.86** | **6.25** |
| | ***Phoxinus phoxinus*** | **Eurasian minnow** | **0.06** | **0.17** | **1.04** |
| | ***Pseudorasbora parva*** | **Topmouth gudgeon** | **0.06** | **0.38** | **2.08** |
| | ***Squalius cephalus*** | **Chub** | **0.009** | **0.17** | **1.04** |
| **Cyprinodontiformes** | ***Gambusia affinis*** | **Mosquitofish** | **0.53** | **3.98** | **16.67** |
| **Clupeiformes** | ***Nematalosa nasus*** | **Gizzard shad** | **0.15** | **0.81** | **4.17** |
| **Perciformes** | ***Sillago sihama*** | **Silver sillago** | **0.04** | **0.28** | **2.08** |
| | ***Ambassis gymnocephalus*** | **Bald glassy** | **0.009** | **0.27** | **2.08** |
| | ***Platycephalus indicus*** | **Bartail flathead** | **0.008** | **0.26** | **1.04** |
| **Spariformes** | ***Acanthopagrus latus*** | **Yellowfin seabream** | **0.02** | **0.39** | **2.08** |
| **Phylum Chordata** | | | **0.003** | **0.12** | **1.04** |
| **Total** | | | **100** | **100** | **-** |

**Table S4** Frequency of occurrence of taxa by the roosting groups based on 18S rDNA.

| Taxa | Roosting groups | | | | All |
|---|---|---|---|---|---|
| | GW10 MP | GW21 MP | LMC (Jan) | LMC (Mar) | |
| **Actinopterygii** | **100** | **100** | **100** | **100** | **100** |
| **Malacostraca** | **96.61** | **37.50** | **87.50** | **25.93** | **73.64** |
| Malacostraca (Class) | 6.78 | 0 | 0 | 0 | 3.64 |
| Decapoda (Order) | 94.92 | 37.5 | 87.5 | 25.93 | 72.73 |
| Atyidae (Family) | 44.07 | 0 | 6.25 | 0 | 24.55 |
| *Penaeus* spp. | 44.07 | 0 | 6.25 | 3.70 | 25.45 |
| *Neomysis* spp. | 1.69 | 0 | 0 | 0 | 0.91 |
| **Vertebrata** | **25.42** | **0** | **18.75** | **18.52** | **20.91** |
| **Platyhelminthes** | **35.59** | **12.5** | **31.25** | **66.67** | **40.91** |
| **Zooplankton** | **20.34** | **0** | **25** | **14.81** | **18.18** |
| **Annelida** | **33.90** | **12.5** | **12.5** | **11.11** | **23.64** |
| **Sponge** | **1.69** | **0** | **0** | **14.81** | **4.55** |
| **Arachnid** | **0** | **25** | **0** | **3.70** | **2.73** |
| **Insecta** | **0** | **0** | **0** | **11.11** | **2.73** |
| **Cnidaria** | **1.69** | **0** | **6.25** | **0** | **1.82** |
| **Nematoda** | **6.78** | **0** | **6.25** | **14.81** | **8.18** |

**Table S5a** Relative read abundances of taxa by the roosting groups based on 18S rDNA.

| Taxa | Roosting groups | | | | All |
|---|---|---|---|---|---|
| | GW10 MP | GW21 MP | LMC (Jan) | LMC (Mar) | |
| **Actinopterygii** | **77.00** | **98.11** | **71.71** | **63.91** | **74.55** |
| **Malacostraca** | **21.33** | **0.53** | **22.97** | **1.55** | **15.20** |
| Malacostraca (Class) | 0.02 | 0 | 0 | 0 | 0.01 |
| Decapoda (Order) | 17.05 | 0.53 | 22.26 | 1.45 | 12.78 |
| Atyidae (Family) | 0.62 | 0 | 0.03 | 0 | 0.34 |
| *Penaeus* spp. | 3.65 | 0 | 0.67 | 0.10 | 2.08 |
| *Neomysis* spp. | 0.002 | 0 | 0 | 0 | 0.001 |
| **Vertebrata** | **0.12** | **0** | **0.03** | **0.18** | **0.11** |
| **Platyhelminthes** | **0.57** | **0.79** | **2.91** | **31.42** | **8.50** |
| **Zooplankton** | **0.14** | **0** | **0.51** | **0.27** | **0.21** |
| **Annelida** | **0.77** | **0.03** | **1.86** | **0.26** | **0.75** |
| **Sponge** | **0.02** | **0** | **0** | **0.74** | **0.19** |
| **Arachnid** | **0** | **0.53** | **0** | **0.63** | **0.19** |
| **Insecta** | **0** | **0** | **0** | **0.09** | **0.02** |
| **Cnidaria** | **0.004** | **0** | **0.01** | **0** | **0.004** |
| **Nematoda** | **0.05** | **0** | **0.01** | **0.93** | **0.26** |

**Table S5b** Relative read abundances of dietary taxa (without Platyhelminthes) by the roosting groups based on 18S rDNA.

| Taxa | Roosting groups | | | | All |
|---|---|---|---|---|---|
| | GW10 MP | GW21 MP | LMC (Jan) | LMC (Mar) | |
| **Actinopterygii** | **77.31** | **98.84** | **73.12** | **94.19** | **82.41** |
| **Malacostraca** | **21.58** | **0.56** | **23.07** | **1.66** | **15.38** |
| Malacostraca (Class) | 0.015 | 0 | 0 | 0 | 0.008 |
| Decapoda (Order) | 17.22 | 0.56 | 22.37 | 1.56 | 12.91 |
| Atyidae (Family) | 0.62 | 0 | 0.031 | 0 | 0.34 |
| *Penaeus* spp. | 3.72 | 0 | 0.67 | 0.10 | 2.12 |
| *Neomysis* spp. | 0.002 | 0 | 0 | 0 | 0.001 |
| **Vertebrata** | **0.12** | **0** | **0.03** | **0.22** | **0.12** |
| **Zooplankton** | **0.14** | **0** | **0.79** | **0.29** | **0.26** |
| **Annelida** | **0.78** | **0.033** | **2.97** | **0.30** | **0.93** |
| **Sponge** | **0.016** | **0** | **0** | **1.50** | **0.38** |
| **Arachnid** | **0** | **0.57** | **0** | **0.63** | **0.20** |
| **Insecta** | **0** | **0** | **0** | **0.14** | **0.035** |
| **Cnidaria** | **0.004** | **0** | **0.01** | **0** | **0.004** |
| **Nematoda** | **0.05** | **0** | **0.01** | **1.06** | **0.29** |

**Table S6** Frequency of occurrence of fish species by the roosting groups using 12S rDNA.

| Orders | Genera/Species | Common names | Roosting groups | | | | All |
|---|---|---|---|---|---|---|---|
| | | | GW10 MP | GW21 MP | LMC (Jan) | LMC (Mar) | |
| **Mugiliformes** | ***Mugil cephalus*** | **Grey mullet** | **88.89** | **37.5** | **0** | **31.58** | **59.38** |
| | ***Liza* spp. (Overall)** | **-** | **85.19** | **75** | **0** | **31.58** | **60.42** |
| | *Liza* spp. | - | 79.63 | 75 | 0 | 26.32 | 56.25 |
| | *Liza subviridis* | Greenback mullet | 51.85 | 12.5 | 0 | 10.53 | 32.29 |
| | *Liza affinis* | Eastern keelback mullet | 12.96 | 0 | 0 | 5.26 | 8.33 |
| | ***Valamugil* spp.** | **-** | **7.41** | **0** | **0** | **0** | **4.17** |
| **Cichliformes** | ***Oreochromis* spp. (Overall)** | **Tilapia** | **53.70** | **87.5** | **60** | **100** | **66.67** |
| | *Oreochromis* spp. | - | 33.33 | 75 | 53.33 | 94.74 | 52.08 |
| | *Oreochromis niloticus* | Nile tilapia | 48.15 | 87.5 | 53.33 | 94.74 | 61.46 |
| | ***Coptodon* spp.** | **-** | **20.37** | **50** | **66.67** | **5.26** | **27.08** |
| **Gobiiformes** | ***Rhinogobius giurinus*** | **Barcheek goby** | **9.26** | **0** | **86.67** | **10.53** | **20.83** |
| | ***Pseudogobius taijiangensis*** | **Taijiang fat-nose goby** | **57.41** | **0** | **0** | **0** | **32.29** |
| | ***Glossogobius giuris*** | **Tank goby** | **14.81** | **0** | **6.67** | **5.26** | **10.42** |
| | ***Hemigobius hoevenii*** | **Banded mullet goby** | **25.93** | **0** | **0** | **0** | **14.58** |
| | ***Gobiopterus lacustris*** | **Lacustrine goby** | **11.11** | **12.5** | **0** | **0** | **7.29** |
| | ***Mugilogobius* spp.** | **-** | **7.41** | **0** | **0** | **0** | **4.17** |
| | *Mugilogobius abei* | Estuarine goby | 5.56 | 0 | 0 | 0 | 3.13 |
| | *Mugilogobius chulae* | Yellowstripe goby | 1.85 | 0 | 0 | 0 | 1.04 |
| | ***Favonigobius gymnauchen*** | **Sharp-nosed sand goby** | **1.85** | **0** | **0** | **0** | **1.04** |
| | **Gobiiformes (Order)** | **-** | **5.56** | **0** | **0** | **0** | **3.13** |
| | **Gobiidae (Family)** | **-** | **0** | **0** | **0** | **5.26** | **1.04** |
| **Cypriniformes** | ***Carassius* spp.** | **Crucian carps** | **3.70** | **0** | **6.67** | **15.79** | **6.25** |
| | ***Phoxinus phoxinus*** | **Eurasian minnow** | **1.85** | **0** | **0** | **0** | **1.04** |
| | ***Pseudorasbora parva*** | **Topmouth gudgeon** | **0** | **0** | **13.33** | **0** | **2.08** |
| | ***Squalius cephalus*** | **Chub** | **1.85** | **0** | **0** | **0** | **1.04** |
| **Cyprinodontiformes** | ***Gambusia affinis*** | **Mosquitofish** | **11.11** | **37.5** | **46.67** | **0** | **16.67** |
| **Clupeiformes** | ***Nematalosa nasus*** | **Gizzard shad** | **5.56** | **12.5** | **0** | **0** | **4.17** |
| **Perciformes** | ***Sillago sihama*** | **Silver sillago** | **3.70** | **0** | **0** | **0** | **2.08** |
| | ***Ambassis gymnocephalus*** | **Bald glassy** | **3.70** | **0** | **0** | **0** | **2.08** |
| | ***Platycephalus indicus*** | **Bartail flathead** | **1.85** | **0** | **0** | **0** | **1.04** |
| **Spariformes** | ***Acanthopagrus latus*** | **Yellowfin seabream** | **3.70** | **0** | **0** | **0** | **2.08** |
| **Phylum Chordata** | **Phylum Chordata** | | **1.85** | **0** | **0** | **0** | **1.04** |

**Table S7** Relative read abundance of fish species by the roosting group using 12S rDNA.

| Orders | Genera/Species | Common names | Roosting groups | | | | All |
|---|---|---|---|---|---|---|---|
| | | | GW10 MP | GW21 MP | LMC (Jan) | LMC (Mar) | |
| **Mugiliformes** | ***Mugil cephalus*** | **Grey mullet** | **35.67** | **2.15** | **0** | **4.99** | **21.23** |
| | ***Liza* spp. (Overall)** | **-** | **33.00** | **14.85** | **0** | **7.81** | **21.35** |
| | *Liza* spp. | - | 24.13 | 13.77 | 0 | 2.59 | 15.23 |
| | *Liza subviridis* | Greenback mullet | 5.01 | 1.08 | 0 | 4.72 | 3.84 |
| | *Liza affinis* | Eastern keelback mullet | 3.86 | 0 | 0 | 0.51 | 2.27 |
| | ***Valamugil* spp.** | **-** | **0.04** | **0** | **0** | **0** | **0.02** |
| **Cichliformes** | ***Oreochromis* spp. (Overall)** | **Tilapia** | **16.47** | **67.93** | **16.67** | **85.45** | **34.44** |
| | *Oreochromis* spp. | - | 6.65 | 31.38 | 11.39 | 41.13 | 16.28 |
| | *Oreochromis niloticus* | Nile tilapia | 9.82 | 36.55 | 5.28 | 44.32 | 18.17 |
| | ***Coptodon* spp.** | **-** | **0.93** | **13.51** | **7.36** | **0.15** | **2.83** |
| **Gobiiformes** | ***Rhinogobius giurinus*** | **Barcheek goby** | **0.30** | **0** | **65.07** | **0.32** | **10.40** |
| | ***Pseudogobius taijiangensis*** | **Taijiang fat-nose goby** | **6.27** | **0** | **0** | **0** | **3.53** |
| | ***Glossogobius giuris*** | **Tank goby** | **2.44** | **0** | **6.67** | **0.22** | **2.46** |
| | ***Hemigobius hoevenii*** | **Banded mullet goby** | **1.23** | **0** | **0** | **0** | **0.69** |
| | ***Gobiopterus lacustris*** | **Lacustrine goby** | **0.73** | **0.02** | **0** | **0** | **0.41** |
| | ***Mugilogobius* spp.** | **-** | **0.52** | **0** | **0** | **0** | **0.29** |
| | *Mugilogobius abei* | Estuarine goby | 0.33 | 0 | 0 | 0 | 0.18 |
| | *Mugilogobius chulae* | Yellowstripe goby | 0.19 | 0 | 0 | 0 | 0.11 |
| | ***Favonigobius gymnauchen*** | **Sharp-nosed sand goby** | **0.01** | **0** | **0** | **0** | **0.01** |
| | **Gobiiformes (Order)** | **-** | **0.62** | **0** | **0** | **0** | **0.35** |
| | **Gobiidae (Family)** | **-** | **0** | **0** | **0** | **0.02** | **0.00** |
| **Cypriniformes** | ***Carassius* spp.** | **Crucian carps** | **1.08** | **0** | **1.77** | **1.03** | **1.09** |
| | ***Phoxinus phoxinus*** | **Eurasian minnow** | **0.10** | **0** | **0** | **0** | **0.06** |
| | ***Pseudorasbora parva*** | **Topmouth gudgeon** | **0** | **0** | **0.38** | **0** | **0.06** |
| | ***Squalius cephalus*** | **Chub** | **0.02** | **0** | **0** | **0** | **0.01** |
| **Cyprinodontiformes** | ***Gambusia affinis*** | **Mosquitofish** | **0.15** | **1.42** | **2.07** | **0** | **0.53** |
| **Clupeiformes** | ***Nematalosa nasus*** | **Gizzard shad** | **0.26** | **0.12** | **0** | **0** | **0.15** |
| **Perciformes** | ***Sillago sihama*** | **Silver sillago** | **0.07** | **0** | **0** | **0** | **0.04** |
| | ***Ambassis gymnocephalus*** | **Bald glassy** | **0.02** | **0** | **0** | **0** | **0.01** |
| | ***Platycephalus indicus*** | **Bartail flathead** | **0.01** | **0** | **0** | **0** | **0.01** |
| **Spariformes** | ***Acanthopagrus latus*** | **Yellowfin seabream** | **0.04** | **0** | **0** | **0** | **0.02** |
| **Phylum Chordata** | **Phylum Chordata** | | **0.01** | **0** | **0** | **0** | **0.003** |

**Table S8a** Incidence-based species richness of 18S and 12S ASV by the roosting groups. Sig, significance groups identified by Turkey's test ($p<0.05$).

| | Species richness | | | |
|---|---|---|---|---|
| | 18S | | 12S | |
| | Mean | Sig | Mean | Sig |
| **GW10-MP** | 4.593 | a | 5.148 | a |
| **GW21-MP** | 2 | b | 4 | ab |
| **LMC-Jan** | 3.625 | a | 3.333 | b |
| **LMC-Mar** | 2.444 | b | 3.053 | b |


| | Species richness | | | |
|---|---|---|---|---|
| | 18S | | 12S | |
| | Mean | Sig | Mean | Sig |
| **GW10-MP** | 4.593 | a | 5.148 | a |
| **GW21-MP** | 2 | b | 4 | ab |
| **LMC-Jan** | 3.625 | a | 3.333 | b |
| **LMC-Mar** | 2.444 | b | 3.053 | b |


**Table S8b** Abundance-based dietary diversities of 18S and 12S rDNA by the roosting groups. Sig, significance groups identified by Turkey's test ($p<0.05$).

| | Simpson's dominance index (D) | | | | Shannon's diversity index (H) | | | |
|---|---|---|---|---|---|---|---|---|
| | 18S | | 12S | | 18S | | 12S | |
| | Mean | Sig | Mean | Sig | Mean | Sig | Mean | Sig |
| **GW10-MP** | 0.641 | b | 0.430 | b | 0.744 | a | 1.105 | a |
| **GW21-MP** | 0.831 | a | 0.391 | b | 0.343 | b | 1.090 | ab |
| **LMC-Jan** | 0.473 | c | 0.700 | a | 0.926 | a | 0.594 | c |
| **LMC-Mar** | 0.845 | a | 0.537 | b | 0.315 | b | 0.806 | bc |

**Table S9a** Pairwise PERMANOVA and SIMPER analyses of taxa between the roosting groups using incidence-based Jaccard dissimilarities of 18S rDNA.

| Roosting group | | PERMANOVA | 18S incidence-based SIMPER (% Contribution) | | | | | | |
|---|---|---|---|---|---|---|---|---|---|
| Group I | Group II | p-value | Gobiidae (Family) | Decapoda (Order) | Atyidae (Family) | *Penaeus* spp. | Haplotaxida (Order) | Arachnida (Class) | Sum |
| **GW10-MP** | **GW21-MP** | 0.006 | | 20.37 | | | | 6.11 | 26.48 |
| | **LMC-Jan** | 0.006 | | | 13.64 | 13.98 | | | 27.62 |
| | **LMC-Mar** | 0.006 | 14.59 | 19.55 | 10.23 | 10.64 | 8.45 | | 63.46 |
| **GW21-MP** | **LMC-Jan** | 0.012 | 29.40 | | | | | | 29.40 |
| | **LMC-Mar** | 1 | | | | | | | - |
| **LMC-Jan** | **LMC-Mar** | 0.006 | 25.48 | 23.47 | | | | | 48.95 |

**Table S9b** Pairwise PERMANOVA and SIMPER analyses of taxa between the roosting groups using abundance-based Bray-Curtis dissimilarities of 18S rDNA.

| Roosting group | | PERMANOVA | 18S abundance-based SIMPER (% Contribution) | | | | |
|---|---|---|---|---|---|---|---|
| Group I | Group II | p-value | Actinopterygii (Class) | Gobiidae (Family) | Decapoda (Order) | *Penaeus* spp. | Sum |
| **GW10-MP** | **GW21-MP** | 0.006 | | 1.78 | 23.01 | 4.66 | 29.45 |
| | **LMC-Jan** | 0.006 | 53.70 | 20.71 | | 3.25 | 77.66 |
| | **LMC-Mar** | 0.006 | 69.19 | 1.67 | 20.96 | 4.34 | 96.15 |
| **GW21-MP** | **LMC-Jan** | 0.006 | 49.46 | 28.84 | 18.84 | | 97.14 |
| | **LMC-Mar** | 1 | | | | | - |
| **LMC-Jan** | **LMC-Mar** | 0.006 | 43.67 | 31.65 | 20.15 | | 95.47 |

**Table S10a** Pairwise PERMANOVA and SIMPER analyses of fish species between the roosting groups using incidence-based Jaccard dissimilarities of 12S rDNA.

| Roosting group | | PERMANOVA | 12S Incidence-based SIMPER (% Contribution) | | | | | | | | | |
|---|---|---|---|---|---|---|---|---|---|---|---|---|
| Group I | Group II | p-value | *Mugil cephalus* | *Liza* spp. | *Oreochromis* spp. | *Coptodon* spp. | *Rhinogobius giurinus* | *Pseudogobius taijiangensis* | *Hemigobius hoevenii* | *Pseudorasbora parva* | *Gambusia affinis* | Sum |
| **GW10-MP** | **GW21-MP** | 0.006 | 11.07 | | | | | 9.98 | | | | 21.05 |
| | **LMC-Jan** | 0.006 | 12.96 | 18.88 | | 8.30 | 12.04 | 7.83 | | 1.51 | 6.82 | 68.34 |
| | **LMC-Mar** | 0.006 | 12.59 | 21.47 | 23.83 | | | 9.84 | 4.00 | | | 71.73 |
| **GW21-MP** | **LMC-Jan** | 0.006 | 8.54 | 15.63 | | | 18.61 | | | | | 42.77 |
| | **LMC-Mar** | 0.036 | | | | 16.24 | | | | | 11.12 | 27.36 |
| **LMC-Jan** | **LMC-Mar** | 0.006 | | | 29.33 | 14.03 | 19.64 | | | | 11.07 | 74.08 |

**Table S10b** Pairwise PERMANOVA and SIMPER analyses of fish species between the roosting groups using abundance-based Bray-Curtis dissimilarities of 12S rDNA.

| Roosting group | | PERMANOVA | 12S abundance-based SIMPER (% Contribution) | | | | | | | |
|---|---|---|---|---|---|---|---|---|---|---|
| Group I | Group II | p-value | *Mugil cephalus* | *Liza* spp. | *Oreochromis* spp. | *Coptodon* spp. | *Rhinogobius giurinus* | *Pseudogobius taijiangensis* | *Gambusia affinis* | Sum |
| **GW10-MP** | **GW21-MP** | 0.006 | 24.68 | | 36.28 | | | 3.88 | | 64.84 |
| | **LMC-Jan** | 0.006 | 24.26 | 19.05 | | 3.32 | 25.81 | 3.84 | | 76.28 |
| | **LMC-Mar** | 0.006 | 24.09 | 19.82 | 44.29 | | | 3.67 | | 91.86 |
| **GW21-MP** | **LMC-Jan** | 0.012 | 1.36 | 9.73 | 22.69 | | 32.15 | | | 65.94 |
| | **LMC-Mar** | 0.030 | | 11.72 | | 11.76 | | | 1.28 | 24.77 |
| **LMC-Jan** | **LMC-Mar** | 0.006 | 3.59 | | 53.23 | 3.85 | 31.94 | | 1.07 | 93.68 |

**Table S11** Details on the collection of feces from *Platalea minor* between January and March 2019. Samples with low quality and wrong host species were excluded from the library preparation. Abbreviations: GW, gei wai; LMC, Lok Ma Chau; 18S, 18S rDNA; 12S, 12S rDNA; Jan, January; Mar, March.

| Locations | Dates | Abbreviations | Species | Number of samples used | |
|---|---|---|---|---|---|
| | | | | **18S** | **12S** |
| **Mai Po (gei wai 10)** | 8-Jan-19 | GW10-MP | *P. minor* | 59 | 54 |
| | | | *P. leucorodi* | 1 | 1 |
| **Mai Po (gei wai 21)** | 8-Jan-19 | GW21-MP | *P. minor* | 8 | 8 |
| **Lok Ma Chau** | 3-Jan-19 | LMC-3 Jan | *P. minor* | 10 | 10 |
| | 23-Jan-19 | LMC-23 Jan | *P. minor* | 6 | 5 |
| | 13-Mar-19 | LMC-13 Mar | *P. minor* | 27 | 19 |
| **Total** | | | | **111** | **97** |

**Table S12** Species compositions of the mock communities (MC) 0 to 4. Five different mock communities were prepared from eight species with known taxonomic identities in classes Actinopterygii (n=6), Malacostraca (n=1), and Gastropoda (n=1). A cross 'X' indicated the species was included in the mock community. MC 0 was prepared by mixing equal weight of tissues (wet weight) from different species before DNA extraction. MC 1 to 4 were prepared by mixing fixed amount of DNA from each of the species.

| Taxonomy | | | 5mg tissues per species | 15ng DNA per species | | | |
|---|---|---|---|---|---|---|---|
| Phyla | Classes | Species | MC0 | MC1 | MC2 | MC3 | MC4 |
| Chordata | Actinopterygii | *Nemipterus virgatus* | X | X | X | X | X |
| | | *Etrumeus teres* | X | X | X | X | X |
| | | *Decapterus maruadsi* | X | X | X | X | X |
| | | *Sillago asiatica* | X | X | X | X | X |
| | | *Protosalanx chinensis* | X | X | X | X | X |
| | | *Betta splendens* | | | | X | X |
| Arthropoda | Malacostraca | *Litopenaeus* sp. | X | | X | | X |
| Mollusca | Gastropoda | *Biomphalaria glabrata* | | | | | X |

**Table S13** Read counts of taxa identified in individual 18S mock communities and negative controls. The species used in the mock communities were indicated by green color. The false positives with relative read abundance lower than 0.08%, 0.02%, and 0.002% were indicated by dark chocolate, orange and yellow colors, respectively. Amplicon sequence variances removed after treatment based on negative controls were labeled as 'Yes'. †ND, not detected; ‡Incertae Sedis, the taxonomic group which broader relationships are unknown or undefined; §MAST-12A, a clade of marine stramenopiles; ¶A31, an uncultured lineage in Alveolata; light green, used in the mock community; dark orange, false positive <0.08%; yellow, false positive <0.02%; light yellow, false positive <0.002%.

| Mock species | Taxa detected by 18S rDNA | Mock communities | | | | | Negative controls | | |
|---|---|---|---|---|---|---|---|---|---|
| | | MC0 | MC1 | MC2 | MC3 | MC4 | Neg1 | Neg2 | Removed |
| **Actinopterygii** | **Actinopterygii (Class)** | 273462 | 450090 | 308195 | 323645 | 150688 | 9 | 4 | - |
| ***Nemipterus virgatus*** | | †ND | ND | ND | ND | ND | | | - |
| ***Decapterus maruadsi*** | | ND | ND | ND | ND | ND | | | - |
| ***Sillago asiatica*** | | ND | ND | ND | ND | ND | | | - |
| ***Protosalanx chinensis*** | | ND | ND | ND | ND | ND | | | - |
| ***Etrumeus teres*** | **Clupeidae (Family)** | 90320 | 174996 | 125310 | 127914 | 64046 | 0 | 0 | - |
| ***Betta splendens*** | ***Betta* spp.** | 3 | 3 | 3 | 148336 | 78544 | 0 | 0 | - |
| ***Penaeus* spp.** | ***Penaeus* spp.** | 223338 | 34 | 169445 | 18 | 75402 | 0 | 0 | - |
| ***Biomphalaria glabrata*** | **Heterobranchia (Order)** | 13 | 0 | 8 | 15 | 264862 | 0 | 0 | - |
| | **Vertebrata (Phylum)** | 203 | 460 | 296 | 402 | 153 | 0 | 0 | - |
| | **Mollusca (Phylum)** | 0 | 0 | 0 | 0 | 273 | 0 | 0 | - |
| | **Aves (Class)** | 100 | 106 | 18 | 2 | 11 | 1 | 1 | - |
| | ***Penicillium* spp.** | 19 | 1 | 0 | 0 | 0 | 0 | 0 | - |
| | **Gobiidae (Family)** | 12 | 31 | 17 | 14 | 7 | 2 | 0 | - |
| | **Loliginidae (Family)** | 12 | 119 | 86 | 72 | 54 | 0 | 0 | - |
| | **Decapoda (Order)** | 10 | 35 | 10 | 21 | 7 | 2 | 1 | - |
| | **Octopoda (Order)** | 10 | 33 | 7 | 14 | 6 | 0 | 0 | - |
| | ***Paradilepis* spp.** | 9 | 4 | 0 | 0 | 4 | 0 | 0 | - |
| | **Mammalia (Class)** | 3 | 0 | 2 | 0 | 0 | 0 | 0 | - |
| | **Eukaryota (Kingdom)** | 3 | 69 | 24 | 28 | 11 | 6954 | 40464 | Yes |
| | **Diplostomida (Order)** | 2 | 1 | 0 | 0 | 0 | 0 | 0 | - |
| | **Rhizophydiales (Order)** | 2 | 0 | 0 | 0 | 2 | 0 | 7363 | Yes |
| | **Embryophyta (Class)** | 2 | 0 | 0 | 1 | 0 | 0 | 0 | - |
| | **‡Incertae Sedis (Class)** | 1 | 0 | 0 | 0 | 0 | 5284 | 13 | Yes |
| | ***Navicula* spp.** | 1 | 1 | 0 | 0 | 0 | 0 | 5543 | Yes |

| Mock species | Taxa detected by 18S rDNA | Mock communities | | | | | Negative controls | | |
|---|---|---|---|---|---|---|---|---|---|
| | | MC0 | MC1 | MC2 | MC3 | MC4 | Neg1 | Neg2 | Removed |
| | ***Poterioochromonas*** **spp.** | 1 | 2 | 0 | 0 | 0 | 0 | 0 | - |
| | ***Desmodesmus*** **spp.** | 1 | 0 | 0 | 0 | 0 | 0 | 0 | - |
| | ***Malassezia*** **spp.** | 1 | 2 | 0 | 0 | 0 | 0 | 0 | - |
| | **Parabasalia (Phylum)** | 1 | 0 | 0 | 0 | 0 | 0 | 0 | - |
| | ***Ramularia*** **spp.** | 1 | 0 | 0 | 0 | 0 | 0 | 0 | - |
| | **[§]MAST-12A (Class)** | 0 | 1 | 0 | 0 | 0 | 0 | 4560 | Yes |
| | ***Ciliophora*** **(Phylum)** | 0 | 0 | 0 | 0 | 0 | 2283 | 0 | Yes |
| | ***Salpingoeca*** **spp.** | 0 | 1 | 0 | 0 | 0 | 0 | 0 | - |
| | ***Tiarina*** **spp.** | 0 | 0 | 0 | 0 | 0 | 0 | 10743 | Yes |
| | **Monhysterida (Order)** | 0 | 0 | 0 | 0 | 0 | 1 | 0 | - |
| | **Rhizophydiaceae (Family)** | 0 | 0 | 0 | 0 | 0 | 0 | 9797 | Yes |
| | ***Cyclotella*** **spp.** | 0 | 1 | 0 | 0 | 1 | 11 | 2042 | Yes |
| | **Haptoria (Family)** | 0 | 0 | 0 | 0 | 0 | 705 | 0 | Yes |
| | **Cyprinidae (Family)** | 0 | 1 | 0 | 0 | 0 | 0 | 0 | - |
| | **Schistosomatidae (Family)** | 0 | 1 | 0 | 0 | 0 | 0 | 0 | - |
| | **Hemiptera (Order)** | 0 | 1 | 0 | 0 | 0 | 0 | 0 | - |
| | ***Dileptus*** **spp.** | 0 | 0 | 0 | 0 | 0 | 0 | 4888 | Yes |
| | **Atyidae (Family)** | 0 | 1 | 0 | 0 | 0 | 0 | 0 | - |
| | ***Paruroleptus*** **spp.** | 0 | 0 | 0 | 0 | 0 | 0 | 4100 | Yes |
| | ***Coelastrum*** **spp.** | 0 | 1 | 0 | 0 | 0 | 0 | 0 | - |
| | **Aphelidea (Family)** | 0 | 0 | 1 | 0 | 0 | 26062 | 3387 | Yes |
| | ***Paraphysomonas*** **spp.** | 0 | 0 | 0 | 0 | 0 | 1 | 0 | - |
| | ***Chromulina*** **spp.** | 0 | 0 | 0 | 0 | 0 | 7652 | 0 | Yes |
| | **Ploimida (Order)** | 0 | 0 | 0 | 0 | 0 | 15730 | 1 | Yes |
| | ***Lembadion*** **spp.** | 0 | 0 | 0 | 0 | 0 | 0 | 905 | Yes |
| | ***Trichoderma*** **spp.** | 0 | 2 | 0 | 0 | 0 | 0 | 0 | - |
| | ***Holosticha*** **spp.** | 0 | 0 | 0 | 0 | 2 | 0 | 0 | - |
| | **Cyclophyllidea (Order)** | 0 | 5 | 0 | 0 | 0 | 0 | 0 | - |
| | **[¶]A31 (Order)** | 0 | 0 | 0 | 0 | 0 | 0 | 16491 | Yes |
| | **Chlorophyceae (Class)** | 0 | 0 | 1 | 0 | 0 | 0 | 0 | - |

**Table S14** Read counts of taxa identified in individual 12S mock communities and negative controls. The species used in the mock communities were indicated by green color. The false positives with relative read abundance lower than 0.08%, 0.02% and 0.002% were indicated by dark chocolate, orange and yellow colors, respectively. Amplicon sequence variances (ASVs) removed after treatment based on negative controls were labelled as 'Yes'. †*Sillago* spp. are the top hits in blastn search against NCBI nt database, but hits from multiple orders in the class of Actinopterygii were also detected. ‡*Protosalanx chinensis* is the top hit but hits from multiple genera in the family of Salangidae were also detected. §ND, this non-fish taxa was not detected by using this marker. Please refer to Table S13 for the colorations.

| Mock species | Taxa detected by 12S rDNA | Mock communities | | | | | Negative controls | | Remove |
|---|---|---|---|---|---|---|---|---|---|
| | | MC0 | MC1 | MC2 | MC3 | MC4 | Neg1 | Neg2 | |
| *Nemipterus virgatus* | *Nemipterus* spp. | 25307 | 1680 | 1870 | 2704 | 2173 | 0 | 0 | - |
| *Decapterus maruadsi* | *Decapterus maruadsi* | 136000 | 69431 | 64550 | 66016 | 67518 | 0 | 0 | - |
| *Sillago asiatica* | †Actinopterygii (Class) | 137827 | 222333 | 193904 | 173850 | 199321 | 0 | 0 | - |
| *Protosalanx chinensis* | ‡Salangidae (Family) | 6739 | 7135 | 11379 | 10652 | 10426 | 0 | 0 | - |
| *Etrumeus teres* | *Etrumeus* spp. | 128650 | 107509 | 102495 | 96910 | 103543 | 0 | 0 | - |
| *Betta splendens* | *Betta splendens* | 1 | 2 | 4 | 42997 | 51175 | 0 | 0 | - |
| *Penaeus* spp. | *Litopenaeus* spp. | §ND | | §ND | | §ND | | | - |
| *Biomphalaria glabrata* | *Biomphalaria glabrata* | | | | | §ND | | | - |
| | *Platalea minor* | 10 | 80 | 6 | 24 | 1 | 1 | 3 | - |
| | *Oreochromis* spp. | 8 | 73 | 0 | 16 | 4 | 4 | 4 | - |
| | *Mugil cephalus* | 6 | 9 | 17 | 9 | 6 | 1 | 0 | - |
| | *Liza subviridis* | 4 | 6 | 0 | 3 | 1 | 0 | 1 | - |
| | *Liza* spp. | 3 | 6 | 4 | 6 | 6 | 1 | 0 | - |
| | *Oreochromis niloticus* | 3 | 50 | 1 | 16 | 2 | 2 | 1 | - |
| | *Acanthopagrus latus* | 2 | 0 | 0 | 0 | 0 | 0 | 0 | - |
| | *Pseudogobius taijiangensis* | 0 | 1 | 0 | 0 | 1 | 1 | 0 | - |
| | *Coptodon* spp. | 0 | 1 | 0 | 0 | 0 | 0 | 3 | - |
| | *Liza affinis* | 0 | 0 | 0 | 0 | 0 | 0 | 1 | - |
| | *Hemigobius hoevenii* | 0 | 1 | 0 | 0 | 0 | 1 | 0 | - |
| | *Glossogobius giuris* | 0 | 0 | 0 | 0 | 0 | 1 | 0 | - |
| | *Gambusia affinis* | 0 | 0 | 0 | 1 | 0 | 0 | 0 | - |
| | *Carassius* spp. | 0 | 0 | 0 | 1 | 0 | 0 | 0 | - |
| | *Homo sapiens* | 0 | 0 | 0 | 0 | 0 | 0 | 117 | Yes |
| | *Phoxinus phoxinus* | 0 | 0 | 0 | 0 | 0 | 0 | 1 | - |
| | *Rhinogobius giurinus* | 0 | 0 | 0 | 1 | 3 | 0 | 1 | - |

**Table S15** Taxa identified by 18S rDNA. A total of 185 taxa were identified by 18S rDNA. Non-dietary taxa, such as Aves (*Platalea* spp.) and Mammals (*Homo sapiens*), were excluded, leaving 183 taxa. Apart from the dietary taxa of *Platalea* spp. in classes of Actinopterygii and Malacostraca (order of Decapoda, family of Atyidae, *Neomysis* spp., and *Penaeus* spp.), all other taxa were grouped into 16 larger taxa (categories) based on all available taxonomic information of each taxon. Non-metazoan taxa including Eukaryota, Fungi, Algae, Protozoa, Plant, Fungus-like, and Fornicata were excluded from data analyses. †MAST-12 or MAST-12A, a clade of marine stramenopiles.

| Kingdoms | Phyla | Classes | Orders | Families | Genera | Categories |
|---|---|---|---|---|---|---|
| **Eukaryota** | Vertebrata | Actinopterygii | | | | Actinopterygii |
| **Eukaryota** | Vertebrata | Actinopterygii | Clupeiformes | Clupeidae | | Actinopterygii |
| **Eukaryota** | Vertebrata | Actinopterygii | Cypriniformes | Cyprinidae | | Actinopterygii |
| **Eukaryota** | Vertebrata | Actinopterygii | Gobiiformes | Gobiidae | | Actinopterygii |
| **Eukaryota** | Arthropoda | Malacostraca | | | | Malacostraca |
| **Eukaryota** | Arthropoda | Malacostraca | Decapoda | | | Decapoda |
| **Eukaryota** | Arthropoda | Malacostraca | Decapoda | Atyidae | | Atyidae |
| **Eukaryota** | Arthropoda | Malacostraca | Decapoda | Penaeidae | *Penaeus* | Penaeus |
| **Eukaryota** | Arthropoda | Malacostraca | Mysida | Mysidae | *Neomysis* | Neomysis |
| **Eukaryota** | Vertebrata | | | | | Vertebrata |
| **Eukaryota** | Platyhelminthes | Cestoda | | | | Platyhelminthes |
| **Eukaryota** | Platyhelminthes | Monogenea | | | | Platyhelminthes |
| **Eukaryota** | Platyhelminthes | Trematoda | Opisthorchiida | Heterophyidae | | Platyhelminthes |
| **Eukaryota** | Platyhelminthes | Trematoda | Diplostomida | Schistosomatidae | | Platyhelminthes |
| **Eukaryota** | Platyhelminthes | Catenulida | | Stenostomidae | | Platyhelminthes |
| **Eukaryota** | Platyhelminthes | Cestoda | Cyclophyllidea | | | Platyhelminthes |
| **Eukaryota** | Platyhelminthes | Monogenea | Mazocraeidea | | | Platyhelminthes |
| **Eukaryota** | Platyhelminthes | Rhabditophora | Rhabdocoela | | | Platyhelminthes |
| **Eukaryota** | Platyhelminthes | Trematoda | Diplostomida | | | Platyhelminthes |
| **Eukaryota** | Platyhelminthes | Cestoda | Cyclophyllidea | Gryporhynchidae | Paradilepis | Platyhelminthes |
| **Eukaryota** | Platyhelminthes | | | | | Platyhelminthes |
| **Eukaryota** | Rotifera | Bdelloidea | Adinetida | | | Zooplankton |
| **Eukaryota** | Arthropoda | Maxillopoda | Calanoida | | | Zooplankton |
| **Eukaryota** | Arthropoda | Maxillopoda | Cyclopoida | | | Zooplankton |

| Kingdoms | Phyla | Classes | Orders | Families | Genera | Categories |
|---|---|---|---|---|---|---|
| **Eukaryota** | Rotifera | Monogononta | Ploimida | | | Zooplankton |
| **Eukaryota** | Arthropoda | Ostracoda | Podocopida | | | Zooplankton |
| **Eukaryota** | Annelida | Clitellata | Haplotaxida | | | Annelida |
| **Eukaryota** | Annelida | Polychaeta | Palpata_Incertae_Sedis | | | Annelida |
| **Eukaryota** | Porifera | Demospongiae | | | | Sponge |
| **Eukaryota** | Porifera | Demospongiae | Spongillida | | | Sponge |
| **Eukaryota** | Porifera | | | | | Sponge |
| **Eukaryota** | Arthropoda | Arachnida | | | | Arachnid |
| **Eukaryota** | Arthropoda | Insecta | Hemiptera | | | Insecta |
| **Eukaryota** | Arthropoda | Insecta | Trichoptera | | | Insecta |
| **Eukaryota** | Cnidaria | Myxozoa | | | | Cnidaria |
| **Eukaryota** | Cnidaria | Myxozoa | Bivalvulida | Myxobolidae | | Cnidaria |
| **Eukaryota** | Nematoda | Chromadorea | Monhysterida | | | Nematoda |
| **Eukaryota** | Nematoda | Chromadorea | Rhabditida | | | Nematoda |
| **Eukaryota** | Nematoda | Enoplea | Triplonchida | | | Nematoda |
| **Eukaryota** | Nematoda | | | | | Nematoda |
| **Eukaryota** | | | | | | Eukaryota |
| **Eukaryota** | Ascomycota | Sordariomycetes | Hypocreales | Incertae_Sedis | *Acremonium* | Fungi |
| **Eukaryota** | Ascomycota | Eurotiomycetes | Eurotiales | Aspergillaceae | *Aspergillus* | Fungi |
| **Eukaryota** | Ascomycota | Dothideomycetes | Dothideales | Aureobasidiaceae | *Aureobasidium* | Fungi |
| **Eukaryota** | Ascomycota | Dothideomycetes | Pleosporales | Pleosporaceae | *Bipolaris* | Fungi |
| **Eukaryota** | Ascomycota | Dothideomycetes | Capnodiales | Cladosporiaceae | *Cladosporium* | Fungi |
| **Eukaryota** | Basidiomycota | Agaricomycetes | | | | Fungi |
| **Eukaryota** | Ascomycota | Dothideomycetes | | | | Fungi |
| **Eukaryota** | Chytridiomycota | Incertae_Sedis | | | | Fungi |
| **Eukaryota** | Ascomycota | Sordariomycetes | | | | Fungi |
| **Eukaryota** | Ascomycota | Saccharomycetes | Saccharomycetales | Metschnikowiaceae | Clavispora-Candida_clade | Fungi |
| **Eukaryota** | Ascomycota | Eurotiomycetes | Chaetothyriales | Herpotrichiellaceae | Exophiala | Fungi |
| **Eukaryota** | Ascomycota | Dothideomycetes | Capnodiales | Extremaceae | Extremus | Fungi |
| **Eukaryota** | Opisthokonta | Aphelidea | | | | Fungi |

| Kingdoms | Phyla | Classes | Orders | Families | Genera | Categories |
|---|---|---|---|---|---|---|
| **Eukaryota** | Ascomycota | Eurotiomycetes | Eurotiales | Aspergillaceae | | Fungi |
| **Eukaryota** | Chytridiomycota | Chytridiomycetes | Chytridiales | Chytriomycetaceae | | Fungi |
| **Eukaryota** | Ascomycota | Dothideomycetes | Pleosporales | Cucurbitariaceae | | Fungi |
| **Eukaryota** | Ascomycota | Saccharomycetes | Saccharomycetales | Debaryomycetaceae | | Fungi |
| **Eukaryota** | Ascomycota | Leotiomycetes | Erysiphales | Erysiphaceae | | Fungi |
| **Eukaryota** | Basidiomycota | Tremellomycetes | Filobasidiales | Filobasidiaceae | | Fungi |
| **Eukaryota** | Chytridiomycota | Mesochytriomycetes | Gromochytriales | Gromochytriaceae | | Fungi |
| **Eukaryota** | Ascomycota | Saccharomycetes | Saccharomycetales | Saccharomycetaceae | | Fungi |
| **Eukaryota** | Basidiomycota | Microbotryomycetes | Sporidiobolales | Sporidiobolaceae | | Fungi |
| **Eukaryota** | Ascomycota | Sordariomycetes | Hypocreales | Nectriaceae | Fusarium | Fungi |
| **Eukaryota** | Basidiomycota | Tremellomycetes | Tremellales | Bulleribasidiaceae | Hannaella | Fungi |
| **Eukaryota** | Ascomycota | Saccharomycetes | Saccharomycetales | Saccharomycetaceae | Kazachstania-Candida_clade | Fungi |
| **Eukaryota** | Basidiomycota | Malasseziomycetes | Malasseziales | Malasseziaceae | Malassezia | Fungi |
| **Eukaryota** | Ascomycota | Sordariomycetes | Myrmecridiales | Myrmecridiaceae | Myrmecridium | Fungi |
| **Eukaryota** | Ascomycota | Dothideomycetes | Pleosporales | Leptosphaeriaceae | Neophaeosphaeria | Fungi |
| **Eukaryota** | Basidiomycota | Cystobasidiomycetes | Cystobasidiales | Incertae_Sedis | Occultifur | Fungi |
| **Eukaryota** | Basidiomycota | Agaricomycetes | Agaricales | | | Fungi |
| **Eukaryota** | Ascomycota | Dothideomycetes | Capnodiales | | | Fungi |
| **Eukaryota** | Ascomycota | Sordariomycetes | Hypocreales | | | Fungi |
| **Eukaryota** | Ascomycota | Dothideomycetes | Pleosporales | | | Fungi |
| **Eukaryota** | Chytridiomycota | Chytridiomycetes | Rhizophydiales | | | Fungi |
| **Eukaryota** | Basidiomycota | Tremellomycetes | Tremellales | | | Fungi |
| **Eukaryota** | Ascomycota | Eurotiomycetes | Eurotiales | Aspergillaceae | Penicillium | Fungi |
| **Eukaryota** | Ascomycota | | | | | Fungi |
| **Eukaryota** | Cryptomycota | | | | | Fungi |
| **Eukaryota** | Ascomycota | Dothideomycetes | Capnodiales | Mycosphaerellaceae | Ramularia | Fungi |
| **Eukaryota** | Basidiomycota | Microbotryomycetes | Sporidiobolales | Sporidiobolaceae | Rhodotorula | Fungi |
| **Eukaryota** | Basidiomycota | Agaricomycetes | Agaricales | Schizophyllaceae | Schizophyllum | Fungi |
| **Eukaryota** | Basidiomycota | Ustilaginomycetes | Ustilaginales | Ustilaginaceae | Sporisorium | Fungi |
| **Eukaryota** | Ascomycota | Dothideomycetes | Superstratomycetales | Superstratomycetaceae | Superstratomyces | Fungi |

| Kingdoms | Phyla | Classes | Orders | Families | Genera | Categories |
|---|---|---|---|---|---|---|
| **Eukaryota** | Ascomycota | Eurotiomycetes | Eurotiales | Trichocomaceae | Talaromyces | Fungi |
| **Eukaryota** | Ascomycota | Sordariomycetes | Hypocreales | Hypocreaceae | Trichoderma | Fungi |
| **Eukaryota** | Basidiomycota | Tremellomycetes | Tremellales | Bulleribasidiaceae | *Vishniacozyma* | Fungi |
| **Eukaryota** | Ascomycota | Saccharomycetes | Saccharomycetales | Debaryomycetaceae | *Yamadazyma-Candida_clade* | Fungi |
| **Eukaryota** | Ochrophyta | Diatomea | Coscinodiscophytina | | *Actinocyclus* | Algae |
| **Eukaryota** | Ochrophyta | Diatomea | Coscinodiscophytina | Melosirids | *Aulacoseira* | Algae |
| **Eukaryota** | Chlorophyta | Trebouxiophyceae | Chlorellales | | *Chlorella* | Algae |
| **Eukaryota** | Chlorophyta | Trebouxiophyceae | Incertae_Sedis | | *Choricystis* | Algae |
| **Eukaryota** | Chlorophyta | Chlorophyceae | | | | Algae |
| **Eukaryota** | Ochrophyta | Chrysophyceae | | | | Algae |
| **Eukaryota** | †MAST-12 | †MAST-12A | | | | Algae |
| **Eukaryota** | Chlorophyta | Trebouxiophyceae | | | | Algae |
| **Eukaryota** | Chlorophyta | Ulvophyceae | | | | Algae |
| **Eukaryota** | Chlorophyta | Chlorophyceae | Sphaeropleales | | *Coelastrum* | Algae |
| **Eukaryota** | Cryptomonadales | Cryptophyceae | Cryptomonadales | | *Cryptomonas* | Algae |
| **Eukaryota** | Ochrophyta | Diatomea | Bacillariophytina | Mediophyceae | *Cyclotella* | Algae |
| **Eukaryota** | Chlorophyta | Chlorophyceae | Sphaeropleales | | *Desmodesmus* | Algae |
| **Eukaryota** | Ochrophyta | Diatomea | Bacillariophytina | Bacillariophyceae | *Encyonema* | Algae |
| **Eukaryota** | Ochrophyta | Diatomea | Bacillariophytina | Bacillariophyceae | | Algae |
| **Eukaryota** | Chlorophyta | Chlorophyceae | Chlamydomonadales | | | Algae |
| **Eukaryota** | Chlorophyta | Trebouxiophyceae | Chlorellales | | | Algae |
| **Eukaryota** | Ochrophyta | Chrysophyceae | Chromulinales | | | Algae |
| **Eukaryota** | Chlorophyta | Ulvophyceae | Cladophorales | | | Algae |
| **Eukaryota** | Cryptomonadales | Cryptophyceae | Cryptomonadales | | | Algae |
| **Eukaryota** | Streptophyta | Zygnemophyceae | Desmidiales | Desmidiaceae | | Algae |
| **Eukaryota** | Ochrophyta | Diatomea | Bacillariophytina | Mediophyceae | | Algae |
| **Eukaryota** | Chlorophyta | Chlorophyceae | Sphaeropleales | | | Algae |
| **Eukaryota** | Ochrophyta | Chrysophyceae | Synurales | | Mallomonas | Algae |
| **Eukaryota** | Chlorophyta | Chlorophyceae | Sphaeropleales | | Messastrum | Algae |
| **Eukaryota** | Chlorophyta | Trebouxiophyceae | Chlorellales | | Micractinium | Algae |

| Kingdoms | Phyla | Classes | Orders | Families | Genera | Categories |
|---|---|---|---|---|---|---|
| **Eukaryota** | Chlorophyta | Chlorophyceae | Sphaeropleales | | Monoraphidium | Algae |
| **Eukaryota** | Chlorophyta | Chlorophyceae | Sphaeropleales | | Mychonastes | Algae |
| **Eukaryota** | Ochrophyta | Diatomea | Bacillariophytina | Bacillariophyceae | Navicula | Algae |
| **Eukaryota** | Ochrophyta | Chrysophyceae | Ochromonadales | | Ochromonas | Algae |
| **Eukaryota** | Ochrophyta | Chrysophyceae | Chromulinales | | Oikomonas | Algae |
| **Eukaryota** | Chlorophyta | Trebouxiophyceae | Chlorellales | | Oocystis | Algae |
| **Eukaryota** | Chlorophyta | Chlorophyceae | Sphaeropleales | | | Algae |
| **Eukaryota** | Ochrophyta | Chrysophyceae | Ochromonadales | | Paraphysomonas | Algae |
| **Eukaryota** | Ochrophyta | | | | | Algae |
| **Eukaryota** | Chlorophyta | Trebouxiophyceae | Incertae_Sedis | | Picochlorum | Algae |
| **Eukaryota** | Cryptomonadales | Cryptophyceae | Cryptomonadales | | Plagioselmis | Algae |
| **Eukaryota** | Ochrophyta | Chrysophyceae | Chromulinales | | Poterioochromonas | Algae |
| **Eukaryota** | Chlorophyta | Chlorophyceae | Sphaeropleales | | Scenedesmus | Algae |
| **Eukaryota** | Ochrophyta | Diatomea | Bacillariophytina | Mediophyceae | Stephanodiscus | Algae |
| **Eukaryota** | Ochrophyta | Chrysophyceae | Synurales | | Synura | Algae |
| **Eukaryota** | Chlorophyta | Chlorodendrophyceae | Chlorodendrales | | Tetraselmis | Algae |
| **Eukaryota** | Ochrophyta | Eustigmatophyceae | Eustigmatales | | Trachydiscus | Algae |
| **Eukaryota** | Ciliophora | Intramacronucleata | Conthreep | Oligohymenophorea | Ancistrum | Protozoa |
| **Eukaryota** | Ciliophora | Intramacronucleata | Spirotrichea | Euplotia | Aspidisca | Protozoa |
| **Eukaryota** | Ciliophora | Intramacronucleata | Conthreep | Oligohymenophorea | Carchesium | Protozoa |
| **Eukaryota** | Choanoflagellida | Craspedida | | | | Protozoa |
| **Eukaryota** | Dinoflagellata | Dinophyceae | | | | Protozoa |
| **Eukaryota** | Cercozoa | Glissomonadida | | | | Protozoa |
| **Eukaryota** | Ciliophora | Intramacronucleata | | | | Protozoa |
| **Eukaryota** | Protalveolata | Perkinsidae | | | | Protozoa |
| **Eukaryota** | Ciliophora | Postciliodesmatophora | Heterotrichea | | Condylostoma | Protozoa |
| **Eukaryota** | Ciliophora | Intramacronucleata | Conthreep | Oligohymenophorea | *Cyclidium* | Protozoa |
| **Eukaryota** | Ciliophora | Intramacronucleata | Conthreep | Oligohymenophorea | *Dexiotricha* | Protozoa |
| **Eukaryota** | Cercozoa | Cercomonadidae | | | | Protozoa |
| **Eukaryota** | Ciliophora | Intramacronucleata | Spirotrichea | Choreotrichia | | Protozoa |

| Kingdoms | Phyla | Classes | Orders | Families | Genera | Categories |
|---|---|---|---|---|---|---|
| **Eukaryota** | Apicomplexa | Conoidasida | Coccidia | Eimeriorina | | Protozoa |
| **Eukaryota** | Ciliophora | Intramacronucleata | Litostomatea | Haptoria | | Protozoa |
| **Eukaryota** | Ciliophora | Intramacronucleata | Spirotrichea | Hypotrichia | | Protozoa |
| **Eukaryota** | Ciliophora | Intramacronucleata | Conthreep | Oligohymenophorea | | Protozoa |
| **Eukaryota** | Ciliophora | Intramacronucleata | Conthreep | Phyllopharyngea | | Protozoa |
| **Eukaryota** | Choanoflagellida | Acanthoecida | Stephanoecidae | | | Protozoa |
| **Eukaryota** | Dinoflagellata | Dinophyceae | Peridiniphycidae | Thoracosphaeraceae | | Protozoa |
| **Eukaryota** | Cercozoa | Thecofilosea | Tectofilosida | | Fisculla | Protozoa |
| **Eukaryota** | Apicomplexa | Conoidasida | Coccidia | Eimeriorina | Goussia | Protozoa |
| **Eukaryota** | Ciliophora | Intramacronucleata | Conthreep | Phyllopharyngea | Heliophrya | Protozoa |
| **Eukaryota** | Ciliophora | Intramacronucleata | Spirotrichea | Hypotrichia | Holosticha | Protozoa |
| **Eukaryota** | Ciliophora | Intramacronucleata | Armophorea | Armophorida | Metopus | Protozoa |
| **Eukaryota** | Protalveolata | Perkinsidae | | | | Protozoa |
| **Eukaryota** | Ciliophora | Intramacronucleata | Conthreep | | | Protozoa |
| **Eukaryota** | Ciliophora | Spirotrichea | Euplotida | | | Protozoa |
| **Eukaryota** | Protalveolata | Syndiniales | | | | Protozoa |
| **Eukaryota** | Protalveolata | Perkinsidae | | | *Parvilucifera* | Protozoa |
| **Eukaryota** | Cercozoa | | Euglyphida | Paulinellidae | *Paulinella* | Protozoa |
| **Eukaryota** | Cercozoa | | | | | Protozoa |
| **Eukaryota** | Dinoflagellata | | | | | Protozoa |
| **Eukaryota** | Parabasalia | | | | | Protozoa |
| **Eukaryota** | Cercozoa | Phytomyxea | | | *Plasmodiophora* | Protozoa |
| **Eukaryota** | Ciliophora | Intramacronucleata | Spirotrichea | Hypotrichia | *Pseudourostyla* | Protozoa |
| **Eukaryota** | Cercozoa | Thecofilosea | Cryomonadida | Rhizaspididae | *Rhogostoma* | Protozoa |
| **Eukaryota** | Choanoflagellida | Craspedida | Salpingoecidae | | *Salpingoeca* | Protozoa |
| **Eukaryota** | Ciliophora | Heterotrichea | Heterotrichida | Spirostomidae | *Spirostomum* | Protozoa |
| **Eukaryota** | Ciliophora | Heterotrichea | Heterotrichida | Stentoridae | *Stentor* | Protozoa |
| **Eukaryota** | Ciliophora | Intramacronucleata | Conthreep | Oligohymenophorea | *Stokesia* | Protozoa |
| **Eukaryota** | Ciliophora | Intramacronucleata | Spirotrichea | Hypotrichia | *Stylonychia* | Protozoa |
| **Eukaryota** | Ciliophora | Intramacronucleata | Conthreep | Oligohymenophorea | *Telotrochidium* | Protozoa |

| Kingdoms | Phyla | Classes | Orders | Families | Genera | Categories |
|---|---|---|---|---|---|---|
| **Eukaryota** | Ciliophora | Intramacronucleata | Spirotrichea | Hypotrichia | *Urostyla* | Protozoa |
| **Eukaryota** | Phragmoplastophyta | Embryophyta | | | | Plant |
| **Eukaryota** | Phragmoplastophyta | Zygnematophyceae | Desmidiales | Closteriaceae | *Closterium* | Plant |
| **Eukaryota** | Phragmoplastophyta | Liliopsida | Poales | | | Plant |
| **Eukaryota** | Phragmoplastophyta | Embryophyta | Liliopsida | | | Plant |
| **Eukaryota** | Heterokontophyta | Oomycota | | | | Fungus-like |
| **Eukaryota** | Heterokontophyta | Oomycota | Peronosporales | Peronosporaceae | *Phytophthora* | Fungus-like |
| **Eukaryota** | Heterokontophyta | Oomycota | Peronosporales | Pythiaceae | *Pythium* | Fungus-like |
| **Eukaryota** | Fornicata | Diplomonadida | Giardiinae | | *Giardia* | Fornicata |
| **Eukaryota** | Vertebrata | Mammalia | | | | Mammal |
| **Eukaryota** | Vertebrata | Aves | | | | Aves |

**Table S16** Taxa identified by 12S rDNA. A total of 31 taxa were identified by 12S rDNA. Apart from *Platalea minor* and *Homo sapiens*, all of the 21 taxa were identified to fish species.

| Domains | Phyla | Classes | Orders | Families | Genera | Species |
|---|---|---|---|---|---|---|
| Eukaryota | Chordata | Actinopterygii | Spariformes | Sparidae | *Acanthopagrus* | *Acanthopagrus latus* |
| Eukaryota | Chordata | Actinopterygii | | Ambassidae | *Ambassis* | *Ambassis gymnocephalus* |
| Eukaryota | Chordata | Actinopterygii | Gobiiformes | Gobiidae | | |
| Eukaryota | Chordata | Actinopterygii | Gobiiformes | Gobiidae | *Favonigobius* | *Favonigobius gymnauchen* |
| Eukaryota | Chordata | Actinopterygii | Cyprinodontiformes | Poeciliidae | *Gambusia* | *Gambusia affinis* |
| Eukaryota | Chordata | Actinopterygii | Cypriniformes | Cyprinidae | *Carassius* | |
| Eukaryota | Chordata | Actinopterygii | Cichliformes | Cichlidae | *Coptodon* | |
| Eukaryota | Chordata | Actinopterygii | Mugiliformes | Mugilidae | *Liza* | |
| Eukaryota | Chordata | Actinopterygii | Cichliformes | Cichlidae | *Oreochromis* | |
| Eukaryota | Chordata | Actinopterygii | Mugiliformes | Mugilidae | *Valamugil* | |
| Eukaryota | Chordata | Actinopterygii | Gobiiformes | Gobiidae | *Glossogobius* | *Glossogobius giuris* |
| Eukaryota | Chordata | Actinopterygii | Gobiiformes | Gobiidae | *Gobiopterus* | *Gobiopterus lacustris* |
| Eukaryota | Chordata | Actinopterygii | Gobiiformes | Gobiidae | *Hemigobius* | *Hemigobius hoevenii* |
| Eukaryota | Chordata | Mammalia | Primates | Hominidae | *Homo* | *Homo sapiens* |
| Eukaryota | Chordata | Actinopterygii | Mugiliformes | Mugilidae | *Liza* | *Liza affinis* |
| Eukaryota | Chordata | Actinopterygii | Mugiliformes | Mugilidae | *Liza* | *Liza subviridis* |
| Eukaryota | Chordata | Actinopterygii | Mugiliformes | Mugilidae | *Mugil* | *Mugil cephalus* |
| Eukaryota | Chordata | Actinopterygii | Gobiiformes | Gobiidae | *Mugilogobius* | *Mugilogobius abei* |
| Eukaryota | Chordata | Actinopterygii | Gobiiformes | Gobiidae | *Mugilogobius* | *Mugilogobius chulae* |
| Eukaryota | Chordata | Actinopterygii | Clupeiformes | Clupeidae | *Nematalosa* | *Nematalosa nasus* |
| Eukaryota | Chordata | Actinopterygii | Gobiiformes | | | |
| Eukaryota | Chordata | Actinopterygii | Cichliformes | Cichlidae | *Oreochromis* | *Oreochromis niloticus* |
| Eukaryota | Chordata | Actinopterygii | Cypriniformes | Cyprinidae | *Phoxinus* | *Phoxinus phoxinus* |
| Eukaryota | Chordata | | | | | |
| Eukaryota | Chordata | Aves | Pelecaniformes | Threskiornithidae | *Platalea* | *Platalea minor* |
| Eukaryota | Chordata | Actinopterygii | Perciformes | Platycephalidae | *Platycephalus* | *Platycephalus indicus* |
| Eukaryota | Chordata | Actinopterygii | Gobiiformes | Gobiidae | *Pseudogobius* | *Pseudogobius taijiangensis* |
| Eukaryota | Chordata | Actinopterygii | Cypriniformes | Cyprinidae | *Pseudorasbora* | *Pseudorasbora parva* |
| Eukaryota | Chordata | Actinopterygii | Gobiiformes | Gobiidae | *Rhinogobius* | *Rhinogobius giurinus* |
| Eukaryota | Chordata | Actinopterygii | Perciformes | Sillaginidae | *Sillago* | *Sillago sihama* |
| Eukaryota | Chordata | Actinopterygii | Cypriniformes | Cyprinidae | *Squalius* | *Squalius cephalus* |

**Table S17** All taxa identified by 18S fecal rDNA of black-faced spoonbill. A threshold of 0.1% was applied to 18S rDNA of each sample to remove the false-positive sequences. Abbreviations: RRA, relative read abundance; wPOO, weighted percentage of occurrence; FOO, frequency of occurrence.

| Taxa | %RRA | %wPOO | %FOO |
|---|---|---|---|
| **Actinopterygii** | **58.49** | **10.96** | **100** |
| **Malacostraca** | **12.55** | **13.92** | **73.87** |
| **Malacostraca (Class)** | 0.01 | 0.39 | 3.6 |
| **Decapoda (Order)** | 10.72 | 8 | 72.97 |
| **Atyidae (Family)** | 0.31 | 2.67 | 24.32 |
| ***Penaeus* spp.** | 1.52 | 2.76 | 25.23 |
| ***Neomysis* spp.** | 0.001 | 0.1 | 0.9 |
| **Vertebrata** | **0.08** | **2.76** | **25.23** |
| **Zooplankton** | **0.07** | **1.97** | **18.02** |
| **Platyhelminthes** | **5.45** | **4.44** | **40.54** |
| **Annelida** | **0.24** | **2.57** | **23.42** |
| **Sponge** | **0.03** | **0.49** | **4.5** |
| **Arachnid** | **0.06** | **0.3** | **2.7** |
| **Insecta** | **0.02** | **0.3** | **2.7** |
| **Cnidaria** | **0.004** | **0.2** | **1.8** |
| **Nematoda** | **0.04** | **0.89** | **8.11** |
| **Eukaryota** | **2.2** | **8.59** | **78.38** |
| **Fungi** | **1.86** | **8.49** | **77.48** |
| **Algae** | **2.14** | **7.7** | **70.27** |
| **Protozoa** | **0.62** | **5.43** | **49.55** |
| **Plant** | **0.93** | **4.44** | **40.54** |
| **Fungus-like** | **0.13** | **1.97** | **18.02** |
| **Fornicata** | **0.002** | **0.1** | **0.9** |
| **Mammal** | **0.87** | **3.36** | **30.63** |
| **Aves** | **14** | **10.17** | **92.79** |
| **Filtered** | **0.21** | **10.96** | **-** |
| **Total** | **100** | **100** | **-** |

**Table S18** All taxa identified by 12S fecal rDNA of black-faced spoonbill. A threshold of 0.05% was applied to 12S rDNA of each sample to remove the false-positive sequences. Abbreviations: RRA, relative read abundance; wPOO, weighted percentage of occurrence; FOO, frequency of occurrence.

| Orders | Genera/Species | Common names | %RRA | %wPOO | %FOO |
|---|---|---|---|---|---|
| **Mugiliformes** | ***Mugil cephalus*** | **Grey mullet** | **17.64** | **9.40** | **59.79** |
| | ***Liza* spp. (All)** | **-** | **16.32** | **15.40** | **60.82** |
| | *Liza* spp. | - | 11.64 | 8.91 | 56.70 |
| | *L. subviridis* | Greenback mullet | 2.73 | 5.19 | 32.99 |
| | *L. affinis* | Eastern keelback mullet | 1.95 | 1.30 | 8.25 |
| | ***Valamugil* spp.** | **-** | 0.01 | 0.65 | 4.12 |
| **Cichliformes** | ***Oreochromis* spp. (All)** | **Tilapia** | **24.76** | **17.67** | **65.98** |
| | *Oreochromis* spp. | - | 11.60 | 8.10 | 51.55 |
| | *O. niloticus* | Nile tilapia | 13.16 | 9.56 | 60.82 |
| | ***Coptodon* spp.** | **-** | 1.80 | 4.21 | 26.80 |
| **Gobiiformes** | ***Rhinogobius giurinus*** | **Barcheek goby** | **5.32** | **3.24** | **20.62** |
| | ***Pseudogobius taijiangensis*** | **Taijiang fat-nose goby** | **2.25** | **5.19** | **32.99** |
| | ***Glossogobius giuris*** | **Tank goby** | **0.60** | **1.62** | **10.31** |
| | ***Hemigobius hoevenii*** | **Banded mullet goby** | **0.57** | **2.27** | **14.43** |
| | ***Gobiopterus lacustris*** | **Lacustrine goby** | **0.11** | **1.13** | **7.22** |
| | ***Mugilogobius* spp.** | **-** | 0.10 | 0.65 | 4.12 |
| | *M. abei* | Estuarine goby | 0.08 | 0.49 | 3.09 |
| | *M. chulae* | Yellowstripe goby | 0.02 | 0.16 | 1.03 |
| | ***Favonigobius gymnauchen*** | **Sharp-nosed sand goby** | **0.01** | **0.16** | **1.03** |
| | **Gobiiformes (Order)** | **-** | **0.14** | **0.49** | **3.09** |
| | **Gobiidae (Family)** | **-** | **0.003** | **0.16** | **1.03** |
| **Cypriniformes** | ***Carassius* spp.** | **Crucian carps** | **0.43** | **0.97** | **6.19** |
| | ***Phoxinus phoxinus*** | **Eurasian minnow** | **0.05** | **0.16** | **1.03** |
| | ***Pseudorasbora parva*** | **Topmouth gudgeon** | **0.02** | **0.32** | **2.06** |
| | ***Squalius cephalus*** | **Chub** | **0.01** | **0.16** | **1.03** |
| **Cyprinodontiformes** | ***Gambusia affinis*** | **Mosquitofish** | **0.33** | **2.59** | **16.49** |
| **Clupeiformes** | ***Nematalosa nasus*** | **Gizzard shad** | **0.11** | **0.65** | **4.12** |
| **Perciformes** | ***Sillago sihama*** | **Silver sillago** | **0.04** | **0.32** | **2.06** |
| | ***Ambassis gymnocephalus*** | **Bald glassy** | **0.01** | **0.32** | **2.06** |
| | ***Platycephalus indicus*** | **Bartail flathead** | **0.01** | **0.16** | **1.03** |
| **Spariformes** | ***Acanthopagrus latus*** | **Yellowfin seabream** | **0.02** | **0.32** | **2.06** |
| **Phylum Chordata** | | | **0.004** | **0.16** | **1.03** |
| ***Primates*** | ***Homo sapiens*** | **Human** | **0.02** | **0.16** | **1.03** |
| **Pelecaniformes** | ***Platalea minor*** | **Black-faced Spoonbill** | **29.32** | **15.72** | **100** |
| **Filtered** | | | **0.01** | **15.72** | **-** |
| **Total** | | | **100** | **100** | **-** |

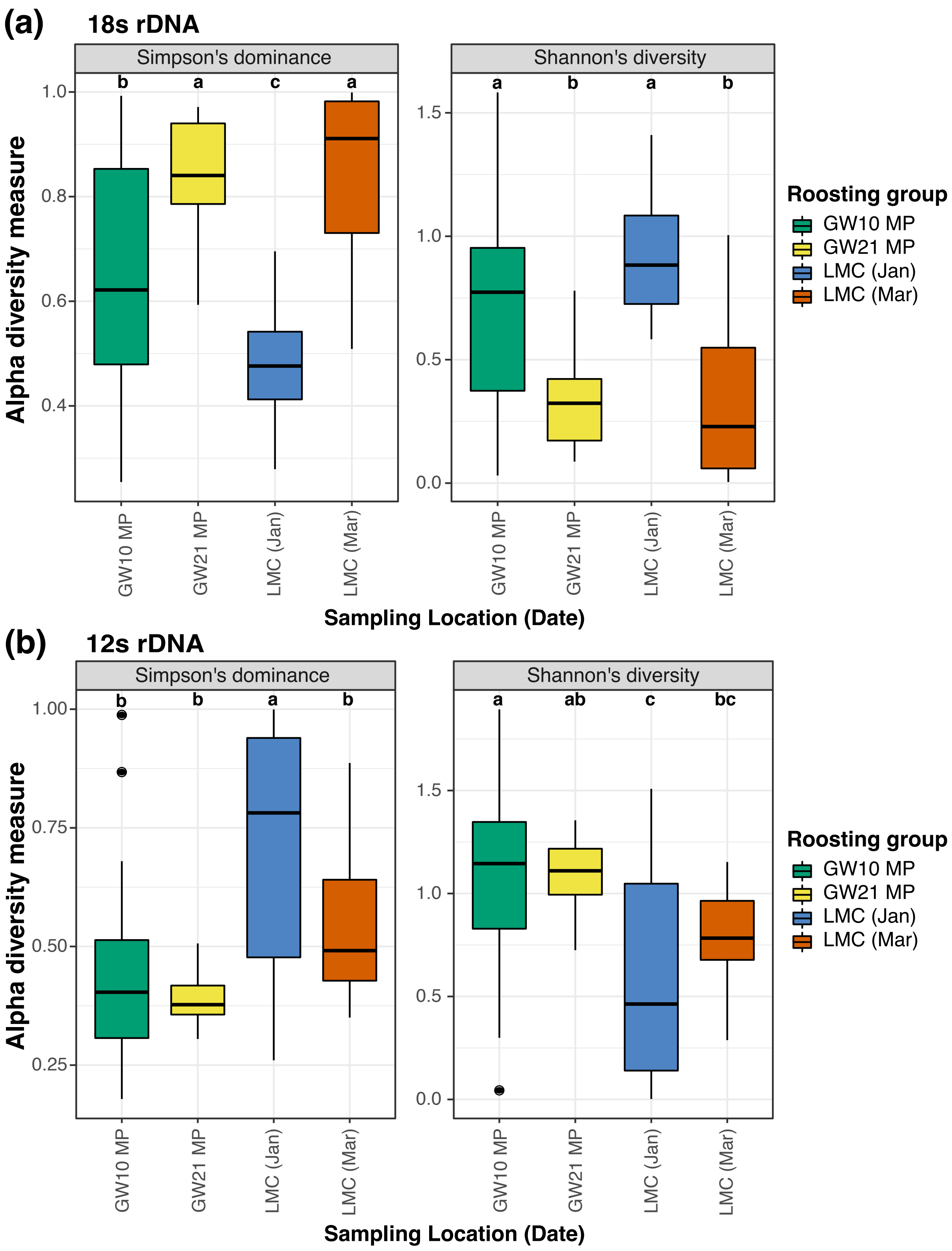


**Figure S1** Abundance-based diversity measures of the dietary taxa in *Platalea minor* based on the (a) 18S and (b) 12S rDNA dataset (n=110 and 96 respectively). The box represents the interquartile range. The line inside the box indicates the median. The upper whiskers extend to the highest value within 1.5× the interquartile range, and the lower whiskers extend to the lowest value within 1.5× the interquartile range. Black dots are outliers. Letters at the top of the boxes indicate significantly different groups. Please see Figure 3 for abbreviations and Table S8b for descriptions of alpha-diversity indices.

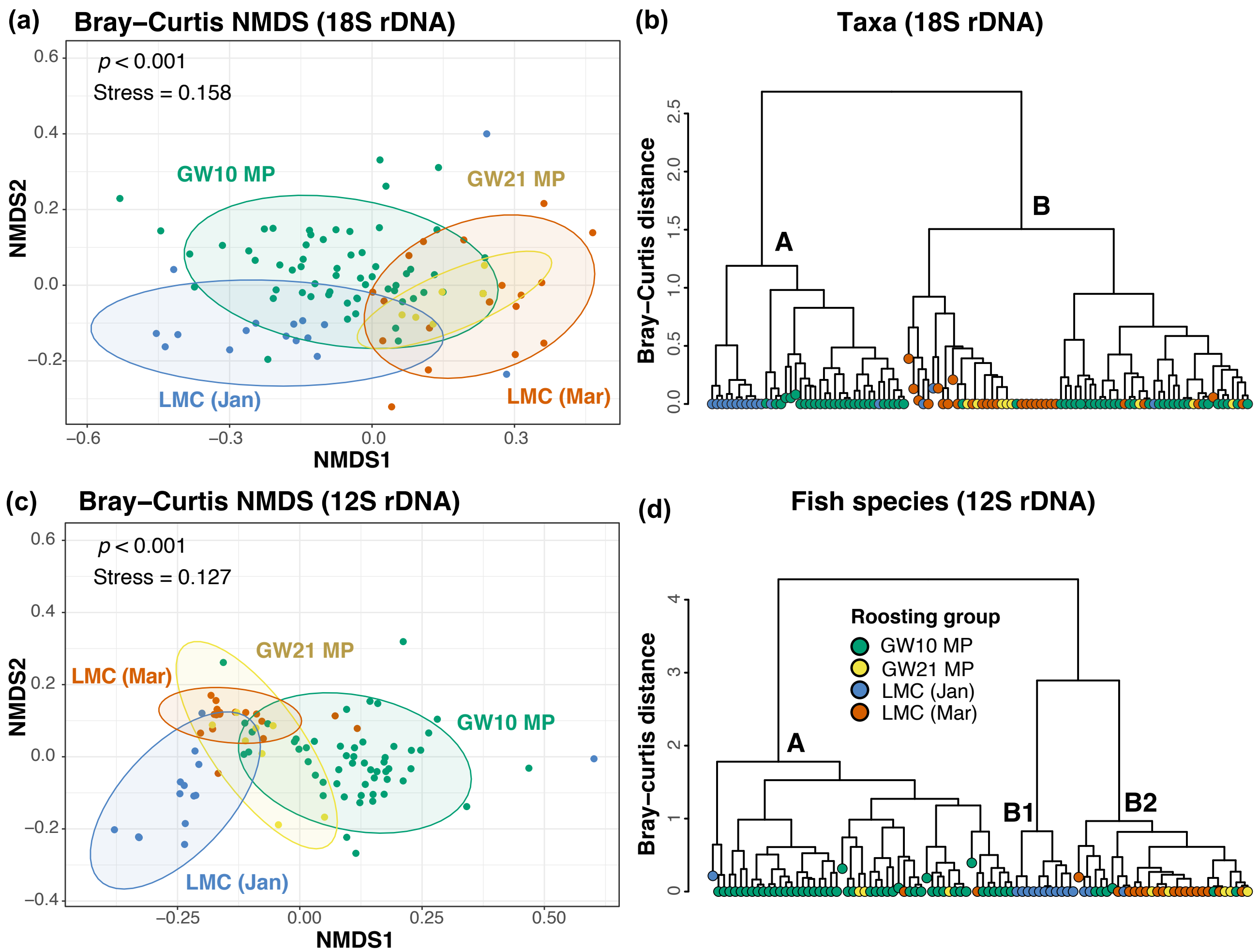


**Figure S2** Abundance-based Bray-Curtis dissimilarities of the dietary taxa compositions between different roosting groups of *Platalea minor*. Relative read abundance of each taxon was fourth root transformed before calculating the dissimilarity distance. (a, c) Nonmetric multidimensional scaling (NMDS) and (b, d) hierarchical clustering dendrograms showed the differences in dietary compositions of taxa (a, b) and fish species (c, d) between the roosting groups identified using 18S and 12S rDNA markers, respectively. Stress level of NMDS analysis and statistically significant differences examined by permutational ANOVA test ($p<0.001$) were indicated. Please see Figure 3 for abbreviations and Table S9b, S10b for SIMPER analysis.
